# Evolving network representation learning based on random walks

**DOI:** 10.1007/s41109-020-00257-3

**Published:** 2020-03-18

**Authors:** Farzaneh Heidari, Manos Papagelis

**Affiliations:** York University, Toronto, M3J1P3 ON Canada

**Keywords:** Network representation learning, Evolving networks, Dynamic random walks, Dynamic graph embedding

## Abstract

Large-scale network mining and analysis is key to revealing the underlying dynamics of networks, not easily observable before. Lately, there is a fast-growing interest in learning low-dimensional continuous representations of networks that can be utilized to perform highly accurate and scalable graph mining tasks. A family of these methods is based on performing random walks on a network to learn its structural features and providing the sequence of random walks as input to a deep learning architecture to learn a network embedding. While these methods perform well, they can only operate on static networks. However, in real-world, networks are evolving, as nodes and edges are continuously added or deleted. As a result, any previously obtained network representation will now be outdated having an adverse effect on the accuracy of the network mining task at stake. The naive approach to address this problem is to re-apply the embedding method of choice every time there is an update to the network. But this approach has serious drawbacks. First, it is inefficient, because the embedding method itself is computationally expensive. Then, the network mining task outcome obtained by the subsequent network representations are not directly comparable to each other, due to the randomness involved in the new set of random walks involved each time. In this paper, we propose EvoNRL, a random-walk based method for learning representations of evolving networks. The key idea of our approach is to first obtain a set of random walks on the current state of network. Then, while changes occur in the evolving network’s topology, to dynamically update the random walks in reserve, so they do not introduce any bias. That way we are in position of utilizing the updated set of random walks to continuously learn accurate mappings from the evolving network to a low-dimension network representation. Moreover, we present an analytical method for determining the right time to obtain a new representation of the evolving network that balances accuracy and time performance. A thorough experimental evaluation is performed that demonstrates the effectiveness of our method against sensible baselines and varying conditions.

## Introduction

Network science, built on the mathematics of graph theory, leverage network structures to model and analyze pairwise relationships between objects (or people) (Newman 2003). With a growing number of networks — social, technological, biological — becoming available and representing an ever increasing amount of information, the ability to easily and effectively perform *large-scale network mining and analysis* is key to revealing the underlying dynamics of these networks, not easily observable before. Traditional approaches to network mining and analysis inherit a number of limitations. First, networks are typically represented as adjacency matrices, which suffer from high-dimensionality and data sparsity issues. Then, network analysis typically requires domain-knowledge in order to carry out the various steps of network data modeling and processing that is involved, before (multiple iterations of) analysis can take place. An ineffective network representation along with a requirement for domain expertise, render the whole process of network mining cumbersome for non-experts and limits their applicability to smaller networks.

To address the aforementioned limitations, there is a growing interest in learning *low-dimensional representations of networks*, also known as *network embeddings*. These representations are learned in an agnostic way (without domain-expertise) and have the potential to improve the performance of many downstream network mining tasks that now only need to operate in lower dimensions. Example tasks include *node classification*, *link prediction* and *graph reconstruction* (Wang et al. 2016), to name a few. Network representation learning methods are typically based on either a *graph factorization* or a *random-walk* based approach. The graph factorization ones (e.g., GraRep (Cao et al. 2015), TADW (Yang et al. 2015), HOPE (Ou et al. 2016)) are known to be memory intensive and computationally expensive, so they don’t scale well. On the other hand, random-walk based methods (e.g., DeepWalk (Perozzi et al. 2014), node2vec (Grover and Leskovec 2016)) are known to be able to scale to large networks. A comprehensive coverage of the different methods can be found in the following surveys (Cai et al. 2018; Hamilton et al. 2017; Zhang et al. 2018).

A major shortcoming of these network representation learning methods is that they can only be applied on *static networks*. However, in real-world, networks are continuously evolving, as nodes and edges are added or deleted over time. As a result, any previously obtained network representation will now be outdated having an adverse effect on the accuracy of the data mining task at stake. In fact, the more significant the network topology changes are, the more likely it is for the mining task to perform poorly. One would expect though that network representation learning should account for continuous changes in the network, in an online mode. That way, (i) the low-dimensional network representation could continue being employed for downstream data mining tasks, and (ii) the results of the mining tasks obtained by the subsequent network representations would be comparable to each other. Going one step further, one would expect that while obtaining the network representation at any moment is possible, the evolving network representation learning framework suggest the best time to obtain the representation based on the upcoming changes in the network.

The main objective of this paper is to develop methods for *learning representations of evolving networks*. The focus of our work is on random-walk based methods that are known to scale well. The naive approach to address this problem is to re-apply the random-walk based network representation learning method of choice every time there is an update to the network. But this approach has serious drawbacks. First, it will be very inefficient, because the embedding method is computationally expensive and it needs to run again and again. Then, the data mining results obtained by the subsequent network representations are not directly comparable to each other, due to the differences involved between the previous and the new set of random walks, as well as, the non-deterministic nature of the deep learning process itself (see “Background and motivation” section for a detailed discussion). Therefore the naive approach would be inadequate for learning representations of evolving networks.

In contrast to the naive approach, we propose a novel random-walk based method for learning representations of evolving networks. The key idea of our approach is to design efficient methods that are incrementally updating the original set of random walks in such a way that it always respects the changes that occurred in the evolving network. As a result, we are able to continuously learn a new mapping function from the evolving network to a low-dimension network representation, by only updating a small number of random walks required to re-obtain the network embedding. The advantages of this approach are multifold. First, since the changes that occur in the network topology are typically local, only a small number of the original set of random walks will be affected, giving rise to substantial time performance gains. In addition, since the network representation will now be continuously informed, the accuracy performance of the network mining task will be improved. Furthermore, since the original set of random walks is maintained as much as possible, subsequent results of the mining tasks will be comparable to each other. In summary, the major contributions of this work include:

- a systematic analysis that illustrates the instability of the random-walk based network representation methods and motivates our work.
- an algorithmic framework for efficiently maintaining a set of random walks that respect the changes that occur in the evolving network topology. The framework treats random walks as “documents” that are indexed using an open-source distributed indexing and searching library. Then, the index allows for efficient ad hoc querying and update of the collection of random walks in hand.
- a novel algorithm, **EVONRL**, for Evolving Network Representation Learning based on random walks, which offers substantial time performance gains without loss of accuracy. The method is generic, so it can accommodate the needs of different domains and applications.
- an analytical method for determining the right time to obtain a new representation of the evolving network. The method is based on adaptive evaluation of the degree of divergence between the most recent random-walk set and the random-walk set utilized in the most recent network embedding. The method is tunable so it can be adjusted to meet the accuracy/sensitivity requirement of different domains, therefore can provide support for a number of real-world applications.
- a thorough experimental evaluation on synthetic and real data sets that demonstrates the effectiveness of our method against sensible baselines, for a varying range of conditions.

An earlier version of this work appeared in the proceedings of the International Conference on Complex Networks and their Applications 2018 (Heidari and Papagelis 2018). The conference version addressed only the case of *adding new edges*. The current version extends the problem to the cases of *deleting existing edges*, *adding new nodes* and *deleting existing nodes*. In addition, it provides an analytical method that aims to provide support to the decision making process of when to obtain a new network embedding. This decision is critical as it can effectively balance accuracy versus time performance of the method extending its applicability in domains of diverse sensitivity. In addition, it provides further experiments for the additional cases that offer substantial, new insights of the problem’s complexity and the performance of our EVONRL method.

The remainder of this paper is organized as follows: “Background and motivation” section provides background and motivates our problem. “Problem definition” section formalizes the problem of efficiently indexing and maintaining a set of random walks defined on the evolving network and “Algorithmic framework of dynamic random walks” section presents our algorithmic framework for addressing it. Our evolving network representation method and analytical method for obtaining new representations of the evolving network are presented in “Evolving network representation learning” section. “Experimental evaluation” section presents the experimental evaluation of our methods and “Extensions and variants” section discusses interesting variants and future directions. After reviewing the related work in “Related work” section, we conclude in “Conclusions” section.

## Background and motivation

As mentioned earlier, there are many different approaches for static network embedding. A family of these methods is based on performing random walks on a network. Random-walk based methods, inspired by the word2vec’s skip-gram model of producing word embeddings (Mikolov et al. 2013b), try to establish an analogy between *a network* and *a document*. While a document is an ordered sequence of words, a network can effectively be described by a set of random walks (i.e., ordered sequences of nodes). Typical examples of these algorithms include DeepWalk (Perozzi et al. 2014) and node2vec (Grover and Leskovec 2016). In fact, the latter can be seen as a generalization of the former, as node2vec can be configured to behave as DeepWalk. We collectively refer to these methods as **StaticNRL** for the rest of the manuscript. A typical StaticNRL method, is operating in two steps:

(i) a set of random walks, say *walks*, is collected by performing *r* random walks of length *l* starting at each node in the network (typical values are *r*=10,*l*=80).
(ii) *walks* are provided as input to an optimization problem that is solved using variants of Stochastic Gradient Descent using a deep neural network architecture (Bengio et al. 2013). The context size employed in the deep learning phase is *k* (typical value is *k*=5). The outcome is a set of *d*-dimensional representations, one for each node.

These representations are learned in an unsupervised way and can be employed for a number of predictive tasks. It is important to note that there are many possible strategies for performing random walks on nodes of a network, resulting in different learned feature representations and different strategies might work better for specific prediction tasks. The methods we will be presenting in this paper are orthogonal to what features the random walks aim to learn, therefore they can accommodate most of the existing random-walk based network representation learning methods.

### Evaluation of the stability of StaticNRL methods

In this paragraph, we present a systematic evaluation of the stability of the StaticNRL methods, similar to the one presented in (Antoniak and Mimno 2018). The evaluation aims to motivate our approach to address the problem of interest. Intuitively, a *stable* embedding method is one in which successive runs of it on the same network would learn the same (or similar) embedding. Our interest for such an evaluation is stemming from the fact that StaticNRL methods are to a great degree dependent on two random processes: (i) the set of random walks collected, and (ii) the initialization of the parameters of the optimization method. Both factors can be a source of instability for the StaticNRL method.

Comparing two embeddings can happen either by measuring their similarity or by measuring their distance. Let us introduce the following measures of instability:

- *Cosine Similarity*: Cosine similarity is a popular similarity measure for real-valued vector space models. It can also been used to compare two network embeddings using the pairwise cosine similarity on the learned *d*-dimensional representations (Kim et al. 2014; Hamilton et al. 2016). Formally, given the vector representations *n*_*i*_ and $n_{i}^{\prime }$ of the same node *n*_*i*_ in two different network embeddings obtained at two different attempts, their cosine similarity is represented as:
  $$sim(n_{i}, n_{i}^{\prime}) = cos(\theta)=\frac{\mathbf{n_{i}} \cdot \mathbf{n_{i}^{\prime}}}{ \|\mathbf{n_{i}} \|\|\mathbf{n_{i}^{\prime}} \|} $$
  We can extend the similarity to two network embeddings *E* and *E*^′^ by summing and normalizing over all nodes:
  $$sim(E, E^{\prime}) = \frac{\sum_{i \in V}sim\left(n_{i}, n_{i}^{\prime}\right)}{|V|} $$
- *Matrix Distance*: Another possible way is to obtain the distance between two network embeddings by subtracting the matrices that represent the embeddings of all nodes, similarly to the approach followed in (Goyal et al. 2018). Formally, given a graph *G*=(*V*,*E*), a network embedding is a mapping $f: V \rightarrow \mathbb {R}^{d}$, where *d*≪|*V*|. Let $F(V) \in \mathbb {R}^{|V| \times d}$ be the matrix of all node representations. Then, we can define the following distance measure for the two network embeddings *E*, *E*^′^:
  $$distance(E, E^{\prime}) = ||F^{\prime}(V) - F(V)||_F $$

**Experimental scenario**: We design a controlled experiment on two real-world networks, namely Protein-Protein-Interaction (PPI) (Breitkreutz et al. 2007) and a collaboration network, Digital Bibliography Library & Project (dblp) (Yang and Leskovec 2015) that aims to evaluate the effect of the two random processes in the final network embeddings. In these experiments, we have three settings. For each setting, we run StaticNRL on a network (using parameter values: *r*=10,*l*=10,*k*=5) two consecutive times, say *t* and *t*+1, and compute the *cosine similarity* and the *matrix distance* of the two network embeddings *E*^*t*^,*E*^*t*+1^ obtained. We repeat the experiment 10 times and report averages. The three settings are:

- *StaticNRL*: Each run collects independent random walks and random weights are used in the initialization phase.
- *StaticNRL-i*: Each run collects independent random walks but employs the same set of weights for the initialization phase, over all runs. The purpose is to eliminate one of the random processes.
- *StaticNRL-rw-i*: Each run employs the same set of random walks and the same set of weights for the initialization phase, over all runs. The purpose is to eliminate both random processes.

**Fig. 1:**
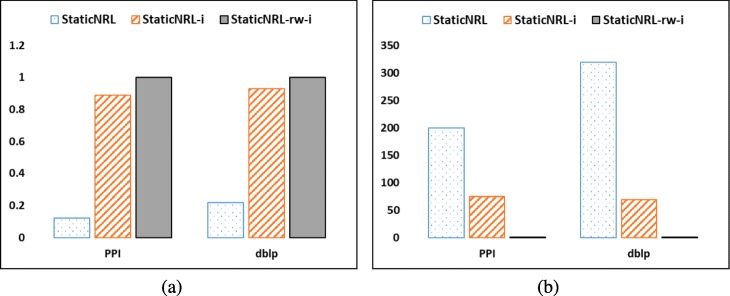
Instability of the StaticNRL methods. Controlled experiments on running StaticNRL multiple times on the same network depict that the network representations learned are not stable, as a result of random initialization and random walks collected. When any of these random processes are fixed, then the network representations learned become more stable. **a** cosine similarity and **b** matrix distance

**Results**: The results of the experiment are shown in Fig. 1a (cosine similarity) and Fig. 1 (matrix distance). They show that the set of random walks and the randomized initialization of the deep learning process have a significant role in moving the embedding despite the fact that there is no actual change in the topology of the network. As a matter of fact, when the same set of random walks and the same initialization is used then consecutive runs of StaticNRL result in the same embedding (as depicted by the *sim*(·,·)=1 in Fig. 1a or *distance*(·,·)=0 in Fig. 1b). However, when the set of random walks is independent or both the random walks and the initialization are independent then substantial differences are illustrated in consecutive runs of the StaticNRL methods.

**Implications**: Let us start by noting that the implications of the experiment is not that StaticNRL is not useful. In fact, it has been shown to work very well. The problem is that while each independent embedding is inherently correct and has approximately same performance in downstream data mining task, these embeddings are not directly comparable to each other. In reality, the embeddings will be approximately equivalent if we are able to rotationally align them — most of similar work in the literature correct this problem by applying an *alignment method* (Hamilton et al. 2016). While alignment methods can bring independent embeddings closer and eliminate the effect of different embeddings, this approach won’t work well in random walk based models. The main reason for that is that as we have showed in the experiment, consecutive runs suffer from instability that is introduced by the random processes. Therefore, in the case of evolving networks (which is the focus of this work), changes that occur in the network topology will not be easily interpretable in the changes observed in the network embedding (since differences might incorporate changes due to the two random processes). However, changes in the evolving network need to be proportional to the changes in the learned network representation. For instance, minor changes in the network topology should cause small changes in the representation, and significant changes in the network topology should cause large changes in the network representation.

**Key idea**: This motivated us to consider learning representations of evolving networks by efficiently maintaining a set of random walks that consistently respect the network topology changes. At the same time, we eliminate the effect of the random processes by, first, preserving, as much as possible, the original random walks that haven’t been affected by the network changes. Then, by initializing the model with a previous run’s initialization (Kim et al. 2014). There are two main advantages in doing so. Changes to the network representations of successive instances of an evolving network will be more interpretable and data mining task results will be more comparable to each other. In addition, it is possible to detect anomalies in the evolving network or extract laws of change in domain-specific networks (e.g., a social network) that explain which nodes are more vulnerable to change, similar to research in linguistics (Hamilton et al. 2016). Furthermore, our framework makes it possible to quantify the importance of any occurring changes in the network topology and therefore obtain a new network representation at an optimal time or when is really needed.

## Problem definition

In “Background and motivation” section, we have established the instability of random walk based methods even when they are repeatedly applied to the same static network. That observation alone highlights the main challenge of employing these methods for learning representations of evolving networks. We have also introduced our key idea to address this problem. Stemming from our key idea, in this Section, we present a few definitions that allow to formally define the problem of interest in this paper.

### **Definition 1**

(*simple random walk or unbiased random walk on a graph*) A *simple random walk* or *unbiased random walk* on a graph is a stochastic process that describes a path in a mathematical space (Pearson 1905), where the random walker transits from its current state (node) to one of its potential new states (neighboring nodes) with an equal probability. For instance, assume a graph *G*=(*V*,*E*) and a source node *v*_0_∈*V*. We uniformly at random select a node *v*_1_ to visit from the set *Γ*(*v*_0_)of all neighbors of *v*_0_. Then, we uniformly at random select a node *v*_2_ to visit from the set *Γ*(*v*_1_) of all neighbors of *v*_1_, and so on. Apparently, the sequence of vertices *v*_0_,*v*_1_,...,*v*_*k*_,... forms *a simple random walk* or *an unbiased random walk* on *G*. Formally, at every step *k*, we have a random variable *X*_*k*_ taking values on *V*, and the random sequence *X*_0_,*X*_1_,...,*X*_*k*_,... is a discrete time stochastic process defined on the state space *V*. Assuming that at time *k* we are at node *v*_*i*_, we select to uniformly at random move to one of its adjacent nodes *v*_*j*_∈*Γ*(*v*_*i*_) based on the following transition probability:

1 $${} p_{v_{i}, v_{j}}=P(X_{k+i} = v_{j} | X_{k} = v_{i}) = \left\{ \begin{array}{ll} \frac{1}{d_{v_{i}}}, \quad if \ (v_{i}, v_{j}) \in E\\ 0, \quad otherwise \end{array}\right.  $$

where $d_{v_{i}}$ is the degree of node *v*_*i*_.

### **Definition 2**

(*biased random walk*) A *biased random walk* is a stochastic process on graph, where the random walker jumps from its current state (node) to one of its potential new states (neighboring nodes) with unequal probability. Formally, assuming that at time *k* we are at node *v*_*i*_, we select to move to one of its adjacent nodes *v*_*j*_∈*Γ*(*v*_*i*_) based on the following transition probability:

2 $${} p_{v_{i}, v_{j}}=P(X_{k+i} = v_{j} | X_{k} = v_{i}) = \left\{ \begin{array}{ll} p, \quad if \ (v_{i}, v_{j}) \in E\\ 0, \quad otherwise \end{array}\right.  $$

where *p* is unequal for each of the neighbours *v*_*j*_∈*Γ*(*v*_*i*_).

### **Definition 3**

(*evolving graph*) Assume a connected, unweighted and undirected graph *G*_*t*_=(*V*_*t*_,*E*_*t*_) where *V*_*t*_ denotes the node set of *G*_*t*_ and *E*_*t*_ denotes the edge set of *G*_*t*_ at time *t*. Since all nodes are connected to at least another node it holds that ∀*u*∈*V*_*t*_ it is *d*_*u*_≥1. Now assume that at time *t*+1 a change occurs in the network topology of *G*_*t*_ forming *G*_*t*+1_=(*V*_*t*+1_,*E*_*t*+1_). This change can occur due to the following events:

- a new edge (*u*^′^,*v*^′^)∉*E*_*t*_ is added in *G*_*t*_; then *E*_*t*+1_=*E*_*t*_∪(*u*^′^,*v*^′^).
- an existing edge (*u*,*v*)∈*E*_*t*_ of *G*_*t*_ is deleted; then, *E*_*t*+1_=*E*_*t*_∖(*u*,*v*).
- a new node *u*^′^∉*V*_*t*_ is added in *G*_*t*_; then *V*_*t*+1_=*V*_*t*_∪*u*^′^.
- an existing node *u*∈*V*_*t*_ of *G*_*t*_ is deleted; then, *V*_*t*+1_=*V*_*t*_∖*u*.

Note that since we have assumed that the graph is connected, the events of *adding a new nodeu*^′^∉*V*_*t*_ in *G*_*t*_ or *deleting an existing nodeu*∈*V*_*t*_ from *G*_*t*_ can be treated as instances of edge addition and edge deletion, respectively. We discuss construction of these cases in “Algorithmic framework of dynamic random walks” section. Over time, nodes and edges are added to and/or deleted from the graph at time *t*^′^=*t*+*i*, *i*∈[1,2,...,+*∞*) forming an *evolving graph*$G_{t}^{\prime }$.

### **Definition 4**

(*a valid set of random walks*) A set of random walks *RW*_*t*_ at time *t* is *valid*, if and only if, every random walk in *RW*_*t*_ is an unbiased random walk on *G*_*t*_.

### **Problem 1**

(*maintaining a valid set of random walks on an evolving network*) Let a connected, unweighted and undirected graph *G*_*t*_=(*V*_*t*_,*E*_*t*_) where *V*_*t*_ denotes the node set of *G*_*t*_ and *E*_*t*_ denotes the edge set of *G*_*t*_ at time *t*. Assume a valid set of random walks *RW*_*t*_ are obtained on *G*_*t*_ at time *t*. As new edges are added to and/or deleted to the evolving graph, at any time *t*^′^=*t*+*i*, *i*∈[1,2,...,+*∞*) forming $G_{t}^{\prime }$, the original set of random walks *RW*_*t*_ will soon be rendered invalid, since many of its random walks will begin introducing a bias. We would like to design and develop methods for efficiently updating any biased random walk in $RW_{t}^{\prime }$ with an unbiased random walk, so that $RW_{t}^{\prime }$ always represents a valid set of random walks of $G_{t}^{\prime }$.

The premise is that if we are able to solve **Problem****1** efficiently, then we will be in a position to obtain an accurate representation of the evolving network at anytime.

## Algorithmic framework of dynamic random walks

In this Section, we describe a general algorithmic framework and novel methods for incrementally updating the set of random walks in reserve, obtained on the original network *G*_*t*_(*V*_*t*_,*E*_*t*_) at time *t*, so that they respect the updated network $G_{t}^{\prime } \left (V_{t}^{\prime }, E_{t}^{\prime }\right)$ at time *t*^′^, where *t*^′^>*t*, and do not introduce any bias. Recall that these are random walks that could have been obtained directly by performing random walks on $G_{t}^{\prime }$. The framework we describe is generic and can be used in any random walk-based embedding method. The first part of the Section presents algorithms for incrementally updating the set of random walks in hand, as edges and/or nodes are added to and/or deleted from the evolving network. The second part, presents an indexing mechanism that supports the efficient storage and retrieval (i.e., query, insert, update, deletion operations) of the set of random walks used for learning subsequent representations of the evolving network. A summary of notations is provided in Table 1.

**Table 1:** Summary of notations used in the dynamic random walk framework

Notations	Descriptions
*G*_*t*_	Network at time *t*
*V*_*t*_	Network’s vertices at time *t*
*E*_*t*_	Network’s edges at time *t*
*G*_*t*+1_	Network at time *t*+1
*E*^+^	A set of the new edges
*V*^+^	A set of the new nodes
$d^{i}_{t}$	Degree of *node*_*i*_ at time *t*
*l*	Length of a random walk
*l*_*sim*_	Length of a simulated random walk
*r*	Number of random walks per node
*RW*_*t*_	A set of random walks at time *t*
*node*_*i*_	A node ∈*V*_*t*_
*e*_*ij*_	A new edge (*node*_*i*_,*node*_*j*_)
*Ind*_*i*_	The position of *node*_*i*_ in a random walk *wk*
*walks*_*i*_	Walks that contain *node*_*i*_

### Incremental update of random walks

Given a network *G*_*t*_=(*V*_*t*_,*E*_*t*_) at time *t*, we employ a standard StaticNRL method^1^ to simulate random walks. This method is configured to perform *r* random walks per node, each of length *l* (default values are *r*=10 and *l*=80). Let *RW*_*t*_ be the set of random walks obtained, where |*RW*_*t*_|=|*V*_*t*_|×*r*. We store the random walks in memory, using a data structure that provides random access to its elements (i.e., a 2-*D*numpy matrix^2^). In practice, each finite-length random walk is stored as a row of a matrix, and each matrix element represents a single node of the network that is traversed by a random walk.

As we monitor changes in the evolving network, there are four distinct events that need to be addressed: i) *edge addition*, ii) *edge deletion*, iii) *node addition*, and iv) *node deletion*. These events can affect the network topology (and the set of random walks in hand) in different ways, therefore they need to be studied separately. First, we provide details of the *edge addition* and *edge deletion* events. This will bring up the challenges that need to be addressed in updating random walks and will introduce our main methods. Then, we visit *node addition* and *node deletion* and show that they can be treated as special cases of *edge addition* and *edge deletion*, respectively.

#### Edge addition

Assume that a single new edge *e*_*ij*_=(*node*_*i*_,*node*_*j*_) arrives in the network at time *t*+1, so *E*_*t*+1_=*E*_*t*_∪(*node*_*i*_,*node*_*j*_). There are two operations that need to take place in order to properly update the set *RW*_*t*_ of the random walks in hand:

- *Operation 1*: contain the new edge to existing random walks in *RW*_*t*_.
- *Operation 2*: discard obsolete parts of random walks of *RW*_*t*_ and replace them with new random walks to form the new *RW*_*t*+1_.

Details of each operation are provided in the next paragraphs.

**Operation 1: contain a new edge in RW** We want to update the set *RW*_*t*_ to contain the new edge (*node*_*i*_,*node*_*j*_). The update should occur in a way that it represents an instance of a possible random walk on *G*_*t*+1_, and at the same time, it preserves the previous set of random walks *RW*_*t*_, as much as possible (to maintain network embedding stability). Note that due to the way that the original set of random walks was obtained, both *node*_*i*_ and *node*_*j*_ will occur in a number of random walks of *RW*_*t*_. We explain the update process for *node*_*i*_; the same process is followed for *node*_*j*_. First, we need to find all the random walks *walks*_*i*_∈*RW*_*t*_ that include *node*_*i*_. Then, we need to update them so as to reflect the existence of the new edge (*node*_*i*_,*node*_*j*_). In practice, the new edge offers a new possibility for each random walk in *G*_*t*+1_ that reaches *node*_*i*_ to traverse *node*_*j*_ in the next step. The number of these random walks that include (*node*_*i*_,*node*_*j*_) depends on the node degree of *node*_*i*_ and it is critical for correctly updating random walks in *RW*. Formally, if the node degree of *node*_*i*_ in *G*_*t*_ is *d*_*t*_ then in *G*_*t*+1_ it will be incremented by one, *d*_*t*+1_=*d*_*t*_+1. Effectively, a random walk that visits *node*_*i*_ in *G*_*t*+1_ would have a probability $\frac {1}{d_{t+1}}$ to traverse *node*_*j*_. This means that if there are *freq*_*i*_ occurrences of *node*_*i*_ in *RW*_*t*_, then $\frac {freq_{i}}{d_{t+1}}$ edges (*node*_*i*_,*node*_*j*_) need to be contained, by setting the next node of *node*_*i*_ to be *node*_*j*_, in the current random walk. If *node*_*i*_ is the last node in a random walk then, there is no need to update the new edge in that random walk.

*Naive approach*: The naive approach to perform the updates is to visit all *freq*_*i*_ occurrences of *node*_*i*_ in *walks*_*i*_∈*RW* and for each of them to decide whether to perform an update of the random walk (or not), by setting the next node to be *node*_*j*_. The decision is based on tossing a biased coin, where with probability $p_{{success}}=\frac {1}{d_{t+1}}$ we update the random walk, and with probability *p*_*failure*_=1−*p*_*success*_ we do not. While this method is accurate, it is not efficient as all occurrences of *node*_*i*_ need to be examined, when only a portion of them needs to be updated.

*Faster approach*: A more efficient way is to find all the *freq*_*i*_ occurrences of *node*_*i*_, and then to uniformly at random sample $\frac {freq_{i}}{d_{t+1}}$ of them and update them by setting the next node to be *node*_*j*_. While this method will be faster than the naive approach, it still resides on finding all the *freq*_*i*_ occurrences of *node*_*i*_ in the set of random walks *RW*, which is an expensive operation. We will soon describe how this method can be accelerated by using an efficient indexing library that allows for fast querying and retrieval of all occurrences a node in random walks.

**Operation 2: replace obsolete random walks** Once a new edge (*node*_*i*_,*node*_*j*_) is contained in an existing random walk, it renders the rest of it obsolete, so it is best to be avoided. Our approach is to replace the remainder of the random walk by simulating a new random walk on the updated network *G*_*t*+1_. The random walk starts at *node*_*j*_ and has a length *l*_*sim*_=*l*−(*Ind*_*i*_+1), where *Ind*_*i*_,0≤*Ind*_*i*_≤*l*−1, is the index of *node*_*i*_ in the random walk that is currently updated. Once updates for *node*_*i*_ have been performed, the updates that are due to *node*_*j*_ are computed and performed.

**Fig. 2:**
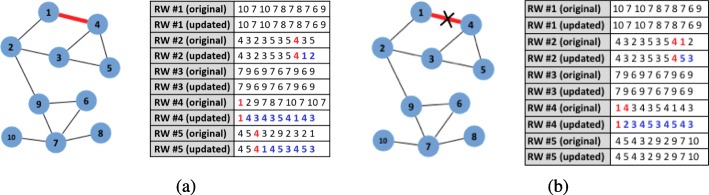
Illustrative example of EVONRL updates for edge addition and edge deletion (colored). **a** Example addition of a new edge (1;4). Random walks in reserve need to be updated to adhere to the change in the network topology. Our method guarantees that the new edge is equally represented in the updated set of random walks. **b** Example deletion of an existing edge (1;4). Random walks in reserve need to be updated to adhere to the change in the network topology. In this example, random walk #2 and #4 traverse edge (1;4) and need to be updated


Figure 2a presents an illustrative example of how updates of random walks work, in the case of a single incoming edge on a simple network. First, a set of random walks *RW*_*t*_ are obtained (say 5 as illustrated by the upper lists of random walks). Let us assume that a new edge (1,4) arrives. Note that now, the degree of node 1 and node 2 will increase by 1 (*d*_*t*+1_=*d*_*t*_+1). Because of the new edge, some random walks need to be updated to account for the change in the topology. To perform the updates, we first search for all occurrences of *i*, *freq*_*i*_. Then, we uniformly at random sample $\frac {freq_{i}}{d_{t+1}} = 2 / 2 = 1$ of them to determine where to contain the new edge. In the example, node 4 is listed after node 1 (i.e., the second node in the random walk #4 is now updated). The rest of the current random walk is obsolete, so it needs to be replaced. To perform the replacement a new random walk is simulated on the updated network *G*_*t*+1_ that starts at node 4 and has a length of *l*_*sim*_=*l*−(*Ind*_1_+1)=10−(0+1)=9. The same process is repeated for node 4 of the added edge (1,4) (see the updates in random walks #2 and #5, respectively).

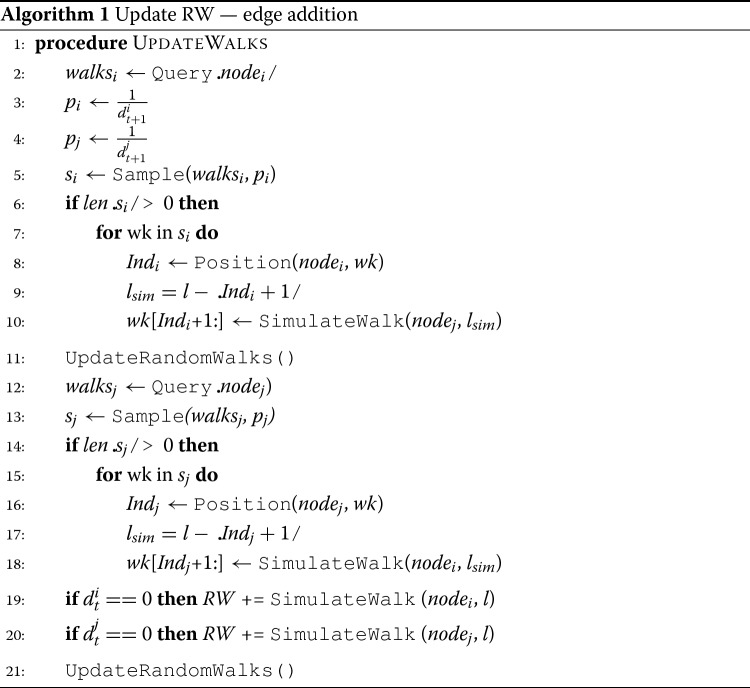


The details of the proposed algorithm are described in Algorithm 1. Lines ?? and ?? of the algorithm invoke a Query operator. This operator is responsible for searching and retrieving information about all the occurrences of *node*_*i*_ in the set of the random walks *RW*_*t*_. In addition, lines ?? and ?? of the algorithm invoke a UpdateRandomWalks operator. This operator is responsible for updating any obsolete random walks of *RW*_*t*_ with the updated ones to form the new valid set of random walks *RW*_*t*+1_, related to *G*_*t*+1_. However, these operators are very computationally expensive, especially for larger networks, and therefore will perform very poorly. In paragraph 1, we describe how these two slow operators, UpdateRandomWalks and Query, can be replaced by similar operators offered off-the-shelf by high performance indexing and searching open-source technologies. In addition, so far, we have relied on maintaining the set of random walks *RW*_*t*_ in memory. However, this is unrealistic for larger networks — while storing a network in memory as an edge list requires *O*(*E*), storing the set of random walks requires *O*(*V*·*r*·*l*) that is typically much larger for sparse networks. The indexing and searching technologies we will employ are very fast and at the same time are designed to scale to very large number of documents. Therefore, they are in position to scale well to very large number of random walks, as we discuss in “Extensions and variants” section.

To accommodate a set of new edges *E*^+^, the same algorithm needs to be applied repeatedly. The main assumption is that edges become available in a temporal order (a stream of edges), which is a common assumption for evolving networks. The premise of our method is that every time, only a small portion of the random walks need to be updated, therefore large performance gains are possible, without any loss in accuracy. In fact, the number of random walks affected depends on the node centrality of the nodes *node*_*i*_ and *node*_*j*_ that form the new edge (*node*_*i*_,*node*_*j*_). While our approach suggests that a new representation is required every time a single change occurs in the network that is not the case in real-world use cases. In fact, in paragraph 1, we provide an analytical method for determining the right time to obtain a new representation of the evolving network. As will see the method is based on an adaptive evaluation of the degree of divergence between the most recent random-walk set and the random-walk set utilized in the most recent network embedding. The method is tunable so it can be adjusted to meet the accuracy/sensitivity requirement of different domains, therefore can provide support for a number of real-world applications. We discuss also the implications of this issue to the time performance of the method in “Experimental evaluation” section.


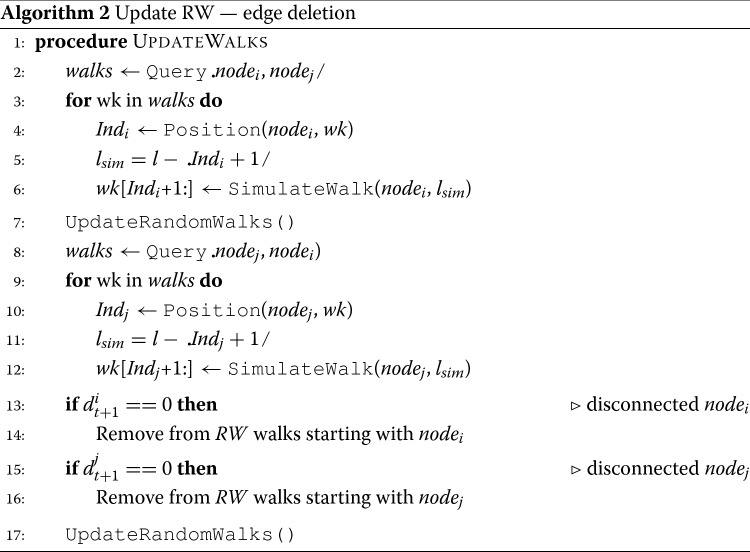


#### Edge deletion

Assume a single existing edge *e*_*ij*_=(*node*_*i*_,*node*_*j*_) is deleted from the network. Similar to edge addition, there are two operations that need to take place:

- *Operation 1*: delete the existing edge from current random walks in *RW*_*t*_ by removing any consecutive occurrence of edge’s endpoints in the set.
- *Operation 2*: discard obsolete parts of random walks of *RW*_*t*_ and replace them with new random walks to form the new *RW*_*t*+1_.

Details of each operation are provided in the next paragraphs.

**Operation 1: delete an existing edge from RW** In edge deletion, unlike with the case of edge addition (where we had to sample over all the occurrences of a specific node), all the walks that have traversed the existing edge (*node*_*i*_,*node*_*j*_) should be modified because all of them are now invalid. Other than that, the rest of the process is similar to that of edge addition. First, all random walks that have occurrences of (*node*_*i*_,*node*_*j*_) and (*node*_*j*_,*node*_*i*_) need to be retrieved. Then, the retrieved random walks need to be modified according to the method described in 1. Algorithm 2 describes this procedure in detail. Figure 2b presents an illustrative example of updates that need to take place due to a single edge deletion. First, a set of random walks are obtained. Let us assume that a new edge (1,4) is deleted, therefore random walks that traverse it, need to be updated. First, we retrieve random walks where node 1 and node 4 occur the one right after the other. For example, in random walk #4 of Fig. 2b, node 4 appears right after 1. Since now that edge doesn’t exist anymore in the network, we need to update the random walk so as to allow an existing neighbor of node 1 to appear after node 4. This action is performed in *operation 2*.

**Operation 2: replace obsolete random walks** This operation is similar to the one in the case of adding a new edge. We just need to replace the remainder of any random walk affected by the *Operation 1* by simulating a new random walk on the updated network *G*_*t*+1_ of the right length. Following up with the running example, to perform the replacement of the obsolete random walk, a new random walk is simulated on network *G*_*t*+1_ that starts at node 1 and has a length of *l*_*sim*_=*l*−(*Ind*_1_+1)=10−(0+1)=9.

*A Note About Disconnected Nodes*: During the process of deleting edges, any of the edge nodes might be disconnected from the rest of the network, forming *isolated nodes*. In that case, all *r* random walks in *RW* that start from an isolated node need to be deleted. In the case that only one of the nodes of a deleted edge becomes isolated, then the simulated random walk is obtained by starting a random walk from the node that remains connected in the network.

#### Node addition

Assume that a new node *node*_*i*_ is added to the network at time *t*+1, so *V*_*t*+1_=*V*_*t*_∪{*node*_*i*_}. Initially, this node forms an *isolated node* (i.e., $d_{i}^{t+1} = 0$) and therefore there is no need to update the set of random walks *RW*. Now, assume that at a later time the node connects to the rest of the network through an edge (*node*_*i*_,*node*_*j*_). In that case, we treat the new edge as described earlier in paragraph 1. In addition to that we need to simulate a set of *r* new random walks, each of length *l*, all of which start from the new node *node*_*i*_ (recall that our original set of random walks consisted of *r* random walks of length *l* for each node in the graph). The newly obtained random walks are appended to *RW*^*t*^ (i.e., it is |*RW*^*t*+1^|=|*RW*^*t*^|+*r*) and are utilized in subsequent network embeddings. There is also a special case where two isolated nodes are connected. In that case we need to simulate *r* random walks of length *l* starting from each node of *node*_*i*_ and *node*_*j*_, respectively and append them to *RW*^*t*^.

#### Node deletion

Assume that an existing node *node*_*i*_ is deleted from the network at time *t*+1, so *V*_*t*+1_=*V*_*t*_∖{*node*_*i*_}. In this case, first we obtain the set of neighbors *Γ*(*node*_*i*_) of *node*_*i*_. For each *node*_*j*_∈*Γ*(*node*_*i*_) there is an edge (*node*_*i*_,*node*_*j*_) in the network that needs to be deleted. We delete each of these edges as described earlier in paragraph 1 and obtain the updated set *RW*. The deletes occur in an arbitrary order, without any side effect. Eventually, this process forms an *isolated node*, which is removed from the graph. Deletion of the isolated node itself doesn’t further affect the set *RW*.

### Efficient storage and retrieval of random walks

The methods of updating random walks presented in the previous paragraph are accurate. However, they depend on operators Query and UpdateRandomWalks that are computationally expensive and cannot scale to larger networks. The most expensive operation is to search the random walks *RW*_*t*_ to find occurrences of *node*_*i*_ and *node*_*j*_ of the new edge (*node*_*i*_,*node*_*j*_). In addition, updates of random walks can be expensive as large number of existing random walks might need to be updated.

**Fig. 3:**
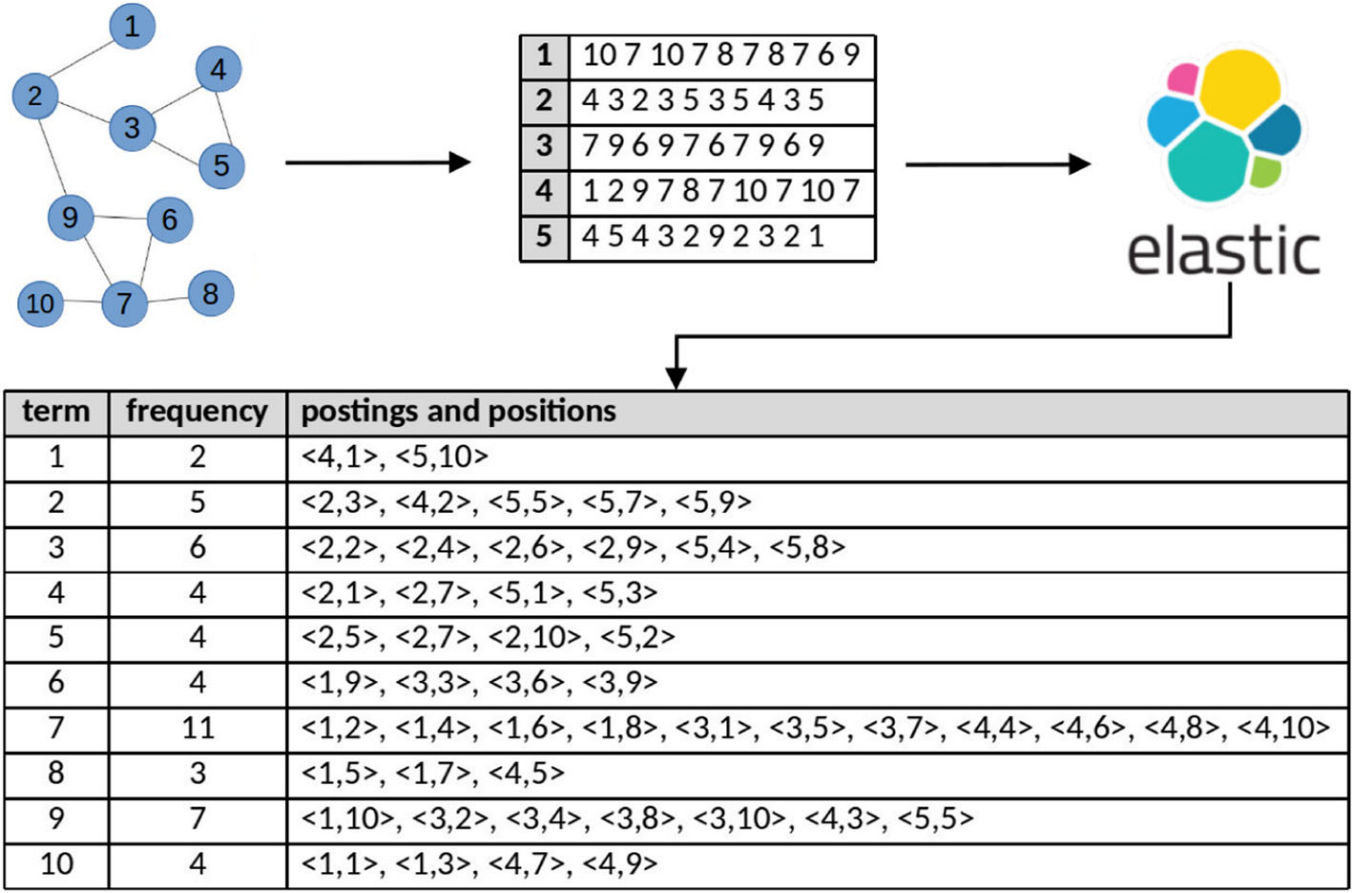
Example inverted random walk index. Given a graph, five random walks are performed. Each random walk is treated as a document and is indexed using an open-source distributed indexing and searching library. The result is an inverted index that provides information about the frequency of any node in the random walks and information about where in the random walk the node is found

To address these shortcomings, our framework of efficiently updating random walks relies on popular open-source indexing and searching technologies. These technologies offer operations for efficiently indexing and searching large collections of documents. For example, they support efficient *full-text search* capabilities where given a query term *q*, all documents in the collection that contain *q* are retrieved. In our framework we treat each random walk as a text “document”. Therefore, each node visited by a random walk would be represented as a text “term”, and all random walks would represent “a collection of documents”. Using this analogy, we build an *inverted random walk index*, *I*^*RW*^. *I*^*RW*^ is an index data structure that stores a mapping from *nodes* (terms) to *random walks* (documents). The purpose of *I*^*RW*^ is to enable *fast querying* of nodes in random walks, and *fast updates* of random walks that can inform Algorithm 1. Figure 3 provides an illustrative example of a small inverted random walk index. In addition, we briefly describe how to create the index and use it in our setting.

*Indexing Random Walks*: We obtain the initial set of random walks *RW*_*t*_ at time *t* by performing random walks on the original network, similarly to the process followed in standard StaticNRL methods. Each random walk is transformed to a document by properly concatenating the ids of the nodes in the walk. For example, a short walk (*x*→*y*→*z*) over nodes *x*, *y* and *z*, will be represented as a document with content “x y z”. These random walks are indexed to create *I*^*RW*^. It is important to note that once an index is available, there is no need to maintain the random walks in memory any more.

*Querying Random Walks*: We rely on the index *I*^*RW*^ to perform any Query operation. Note, however, that there are additional advantages on using an efficient index. Besides searching and retrieving all random walks that contain a specific *node*_*i*_, the index *I*^*RW*^ can be configured to provide more quantities of interest. Specifically, we configure *I*^*RW*^ so that every query retrieves additional information about the frequency of *node*_*i*_,*freq*_*i*_ and the position *Ind*_*i*_ of *node*_*i*_ in a retrieved random walk (see Fig. 3). The first quantity (*freq*_*i*_) is used to determine the number of updates that are required as discussed earlier. The second (*Ind*_*i*_), is used to inform the operator Position in Algorithm 1 (lines ?? and ??). Note that there is a slight variation of how the Query operation is configured in the case of the edge deletion. Recall that in that event we need to retrieve random walks where the two nodes *node*_*i*_ and *node*_*j*_ are found the one right after the other (i.e., they form a step of the random walk). To accommodate this case we just need to configure the Query operation to retrieve all random walks that contain the bigram “ *node*_*i*_*node*_*j*_”. A bigram is a pair of contiguous sequence of words in a document or, following the analogy, a pair of contiguous sequence of nodes in a random walk. The indexing and searching technology we employ can handily support such queries.

*Updating Random Walks*: We rely on the index *I*^*RW*^ for any UpdateRandomWalk operation. An update of a random walk is analogous to an update of a document in the index. In practice, any update of the index *I*^*RW*^ is equivalent to deleting an old random walk and then indexing a new random walk. While querying using an inverted index is a fast process, updating an index is a slower process. Therefore, the performance of our methods is dominated by the number of random walks updates required. Still, our methods would perform multitude of times faster than StaticNRL methods. A detailed analysis of this issue is provided in “Experimental evaluation” section. Following the discussion about the edge deletion/addition, special care is required when these events involve *isolated nodes*. In particular, if a new edge connects a previously isolated node *node*_*i*_ to the network, then *r* new random walks need to be added in the index, each of which starts from *node*_*i*_. The process of indexing the new random walks is similar to the process described in paragraph 1. Similarly, if an edge deletion event resulted in a node *node*_*i*_ being isolated, then all the *r* random walks that start from *node*_*i*_ need to be removed from the index. Removing a random walk from the index is analogous to deleting a document from the index.

**Bulk updates**: Additional optimizations are available as a result of employing an inverted index for the random walks. For example, we can take advantage of *bulk updates*, where the index need only be updated when a number of new edges have arrived. This means that changes of single incoming edges won’t be reflected in *I*^*RW*^ right away. While this optimization has the premise to make our methods faster (since updates occur once in a while), it risks harming its accuracy. In practice, it offers an interesting trade-off between accuracy and time performance that domain-specific applications need to tune. Experiments in “Experimental evaluation” section demonstrate this tradeoff.

## Evolving network representation learning

So far we have described our framework for maintaining an always valid set of random walks *RW*_*t*_ at time *t*. Recall that our final objective is to be able to learn a representation of this evolving network. For the embedding process we resort to the same embedding of standard StaticNRL methods. Below we describe how embeddings of the evolving network are obtained, given a set of random walks *RW*_*t*_. Then, a general strategy for obtaining an embedding only when it is mostly needed.

### Learning embeddings

Given a general network, *G*_*t*_=(*V*_*t*_,*E*_*t*_), our goal is to learn the network representation *f*(*V*_*t*_) using the skip-gram model. *f*(*V*_*t*_) is a |*V*_*t*_|×*d* matrix where *d* is the network representation dimension and each row is the vector representation of a node. At the first time-stamp, the node vector representations (neural network’s weights) are initialized randomly and we use this initialization for other timestamps’ training. The training objective function is to maximize the log-probability of the nodes appearing in the context of the node *n*_*i*_. Context of each node *n*_*i*_ is provided by the valid set of random walks *RW*_*t*_, similarly to the process described in previous work (Perozzi et al. 2014; Grover and Leskovec 2016). Using the approximate objective, skip-gram with negative sampling (Mikolov et al. 2013a), these embeddings are optimized by stochastic gradient decent so that:

3 $$ Pr(\mathit{n_{j}}|\mathbf{n_{i}}) \propto \exp{\left(\mathbf{n_{j}^{T}}\mathbf{n_{i}}\right)}  $$

where *n*_*i*_ is the vector representation of a node *n*_*i*_(*f*(*n*_*i*_)=*n*_*i*_). *Pr*(*n*_*j*_|*n*_*i*_) is the probability of the observation of neighbor node *n*_*j*_, within the window-size given that the window contains *n*_*i*_. In our experiments, we use the gensim implementation of the skip-gram model^3^. We set our context-size to *k*=5 and the number of dimensions to *d*=128, unless otherwise stated.

### Analytical method for determining the timing of a network embedding

EVONRL has the overhead of first indexing the set of initial random walks *RW*. At that time, we randomly initialize the skip-gram model and keep these initialization weights for the learning phase of subsequent times. As new edges/nodes are added/deleted, EVONRL performs the necessary updates as described earlier. At each time step a valid set of random walks is available that can be used to obtain a network embedding. As we show in “Experimental evaluation” section an embedding obtained by our incrementally updated set of random walks effectively represents embeddings obtained by applying a StaticNRL method directly on the updated network. However, while re-embedding the network every time a change occurs in it will result in accurate embeddings, this process is very expensive and risks to render the method non-applicable in real-world scenarios. Therefore, and depending on the domain, it is reasonable to assume that only a limited number of re-embeddings be obtained. This introduces a new problem: *when is the right time to obtain a network embedding*? In fact, this decision process demonstrates an interesting tradeoff between accuracy and time performance of the method proposed. In the rest of the paragraph we introduce two strategies for determining the time to obtain network embeddings.

PERIODIC: This is a sensible baseline where, as the name reveals, obtains embeddings periodically, every *q* time steps. Depending on the sensitivity of the domain we operate on, the period can be shorter or longer. This method is easy to implement, but it is obtaining network embedding being agnostic of the different changes that occur in the network and whether they are significant (or not).

ADAPTIVE: We introduce an analytical method for determining the right timing of obtaining a network embedding. The key idea of the method is to continuously monitor the changes that occur in the network. Then, if significant changes are detected it obtains a new network embedding. In fact, we monitor two conditions, the first is able to detect occurrence of a critical change (e.g., addition of a very important edge) and is based on the idea of *peak detection*; the second is able to evaluate cumulative effects due to a number changes. We discuss the structure of these conditions in the following paragraphs.

*Peak detection*: We start by providing background of a *z*-score. A *z*-score (or standard score) is a popular statistical measure that indicates how many *standard deviations* away an observation is from its *mean*. When the population mean and the population standard deviation are unknown, the standard score may be calculated using the *sample mean* and *sample standard deviation* as estimates of the population values. In that case, the *z*-score of observed values *x* can be calculated from the following formula:

4 $$  z = \frac{x - \hat{x}}{\hat{\sigma}}  $$

where $\hat {x}$ is the mean of the sample and $\hat {\sigma }$ is the standard deviation of the sample.

In our setting, we want to detect when important changes occur in the network, so as to obtain a timely network representation. As we described earlier a good proxy for what consists an important change in a network is the number of random walks that are affected because of the change (edge addition/deletion, node addition/deletion). We can utilize the *z*-score of Eq. (4) to detect *peaks*. A peak or spike is a generic term which describes a sudden increase or outburst in a sequenced data (Barnett and Lewis 1974). In our problem, the number of random walk changes are monitored and peaks represent significant changes in the number of random walks affected. Formally, let *lag* be the number of changes observed in the sample. The observation window is spanning from *t*−*lag* to *t* and we compute the mean of the sample at *t* as *avg*[*t*]. In a similar way, we calculate the standard deviation of the sample at *t* to be *std*[*t*]. Let *N*[*t*] be the observation at time *t* that represents the number of random walks that have been updated due to a network change. Now, given *N*[*t*],*avg*[*t*],*std*[*t*] and a threshold *τ*, a peak occurs at time *t* if the following condition holds:

5 $$  N[t] > \tau \times std[t] + avg[t]  $$

**Fig. 4:**
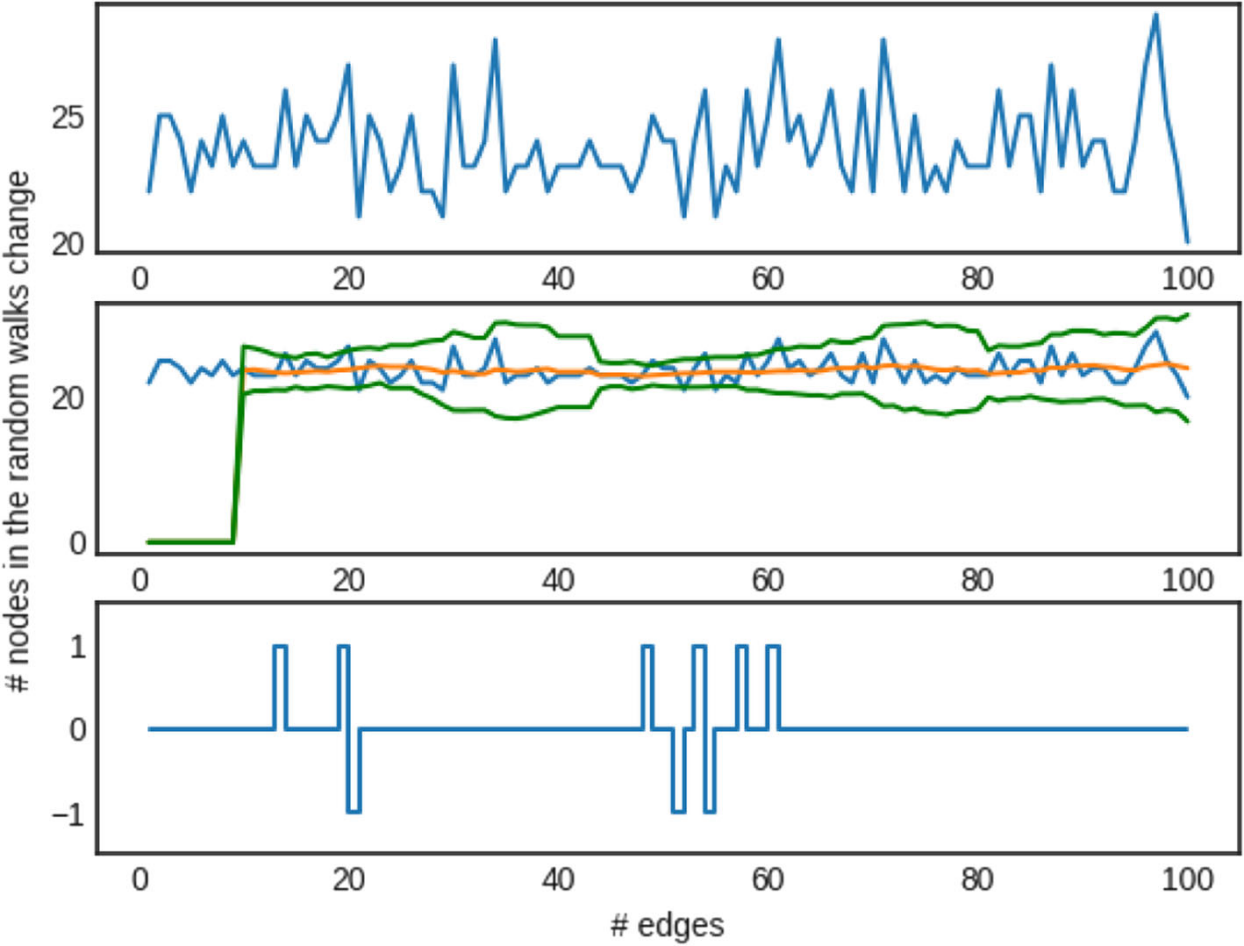
Example peak detection method for the case of adding edges in the *BlogCatalog* network. The upper plot shows the number of random walks that are updated in *RW* as a function of new edges added. It is evident that some edges have a larger effect in *RW* as depicted by higher values. The middle plot, shows the mean (middle almost straight line), as well as the boundaries defined by the current threshold of *τ*×*std* (the two lines above and below the mean line). The bottom plot provides the signal for decision making; every time that the current change at time *t* is outside the threshold it signals that a network embedding should be obtained. In the example this is the case for five times *t*={13,19,48,53,57,60}

If the condition of Eq. (5) holds, then we know that a significant change has occurred and we decide to obtain a new network representation. The details of the procedure are shown in Algorithm 3. Notations used in this algorithm are summarized in Table 2. Figure 4 provides an illustrative example of the peak detection method. In this example we set *lag*=10 and *τ*=3. The figure shows the results of the peak detection method for 100 changes occurring in a network (*BlogCatalog network*, *edge addition*; edges are added one by one and are randomly selected from the potential edges of the network). Our peak detection algorithm detects a total of 6 peaks occurring at *t*={13,19,48,53,57,60}.

**Table 2:** Summary of notations used in decision-making algorithm

Notations	Descriptions
*RW*_*t*_	A set of random walks at time *t*
*RW*_*t*+1_	A set of random walks at time *t*
$N_{t}^{t+1}$	Number of the nodes changed from *t* to *t*+1
$\# RW_{t}^{t+1}$	Number of random walks changed from *t* to *t*+1
*τ*	Threshold where algorithm signals
*lag*	The size of the moving window
*avg*	Moving average of the lag window
*std*	Standard deviation of the lag window


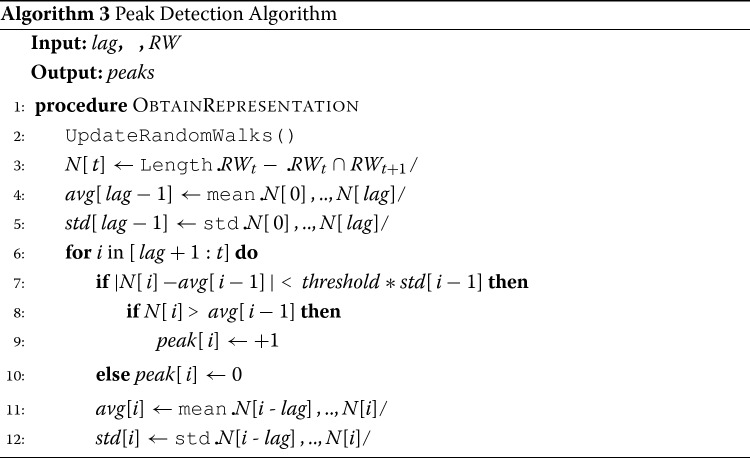


*Cut-off score*: Sometimes, changes in the network can be smooth, without any acute changes. In that case the peak detection method will fail to obtain any embedding as peaks (almost) never occur. To avoid these cases, besides the peak detection method, we employ an additional metric that monitors the cumulative effect of all the changes since the last embedding was obtained. Formally, let *N*[*t*] be the observation at time *t* that represents the number of random walks that have been updated due to a network change. Then, the total number of random walks that have been changed between the time that the last embedding *t*_*old*_ was obtained and the current time *t* is given by:

6 $$ \#RW_{t_{{old}}}^{t} = \sum_{t=t^{old}}^{t} N[t]  $$

Now, given $\#RW_{t_{{old}}}^{t}$ and a threshold *cutoff*, we monitor the following condition:

7 $$  \#RW_{t_{{old}}}^{t} > cutoff  $$

If at any time *t* Eq. (7) holds, then we know that significant cumulative changes have occurred in the network and we decide to obtain a new network representation.

As we show in “Experimental evaluation” section combining both conditions of Eqs. (5) and (7) gives the best results, as it balances locally significant as well as cumulative effect of changes.

## Experimental evaluation

In this Section, we experimentally evaluate the performance of our dynamic random walk framework and EVONRL^4^. In particular, we aim to answer the following questions:

- **Q1 effect of network topology** How the topology of the network affects the number of random walks that need to be updated?
- **Q2 effect of arriving edge importance** How edges of different importance affect the overall random walk update time?
- **Q3 accuracy performance of**EVONRL What is the accuracy performance of EvoNRL compared to the ground truth provided by StaticNRL methods?
- **Q4 classification performance of**EVONRL What is the accuracy performance of EvoNRL in a downstream data-mining task?
- **Q5 time performance of**EVONRL What is the time performance of EvoNRL?
- **Q6 decision-making performance of**EVONRL How well does the strategy of EvoNRL for obtaining network representations work?

**Q1** and **Q2** aim to shed light on the behavior of our generic computational framework for dynamically updating random walks in various settings. **Q3**, **Q4**, **Q5** and **Q6** aim to demonstrate how EVONRL performs. Before presenting the results, we provide details of the computational environment and the data sets employed.

**Environment**: All experiments are conducted on a workstation with 8x Intel(R) Core(TM) i7-7700 CPU @ 3.60GHz and 64GB memory. Python 3.6 is used and the static graph calculations use the state-of-the-art algorithms for the relevant metrics provided by the *NetworkX* network library.

**Data**: For the needs of our experiments both synthetic data and real data sets have been employed.

- *Protein-Protein Interactions (PPI):* We use a subgraph of PPI for Homo Sapiens and use the labels from the preprocessed data used in (Grover and Leskovec 2016). The network consists of 3890 nodes, 76584 edges and 50 different labels.
- *BlogCatalog (*Reza and Huan*):* BlogCatalog is a social network of blogers which each edge indicates a social interaction among them. This network consists of 10312 nodes, 333983 edges and 39 different labels.
- *Facebook Ego Network (*Leskovec and Krevl 2014*):* Facebook ego network is the combined ego network of each node. There is an edge from a node to each of its friends. This network consists of 4039 nodes, 88234 edges.
- *Arxiv HEP-TH (*Leskovec and Krevl 2014*):* Arxiv HEP-TH (high energy physics theory) network is the citation network from e-print Arxiv. If paper *i* cites paper *j*, there is a directed edge from *i* to *j*. This network consists of 27770 nodes, 352807 edges.
- *Synthetic Networks*: We create a set of Watts-Strogatz (Newman 2003) random networks of different sizes (*n*={1000,10000}) and different rewiring probabilities (*p*={0,0.5,1.0}). The rewiring probability is used to create representative *Lattice* (*p*=0), *Small-world* (*p*=0.5) and *Erdos-Reyni* (*p*=1) networks, respectively.

### Q1 effect of network topology

**Fig. 5:**
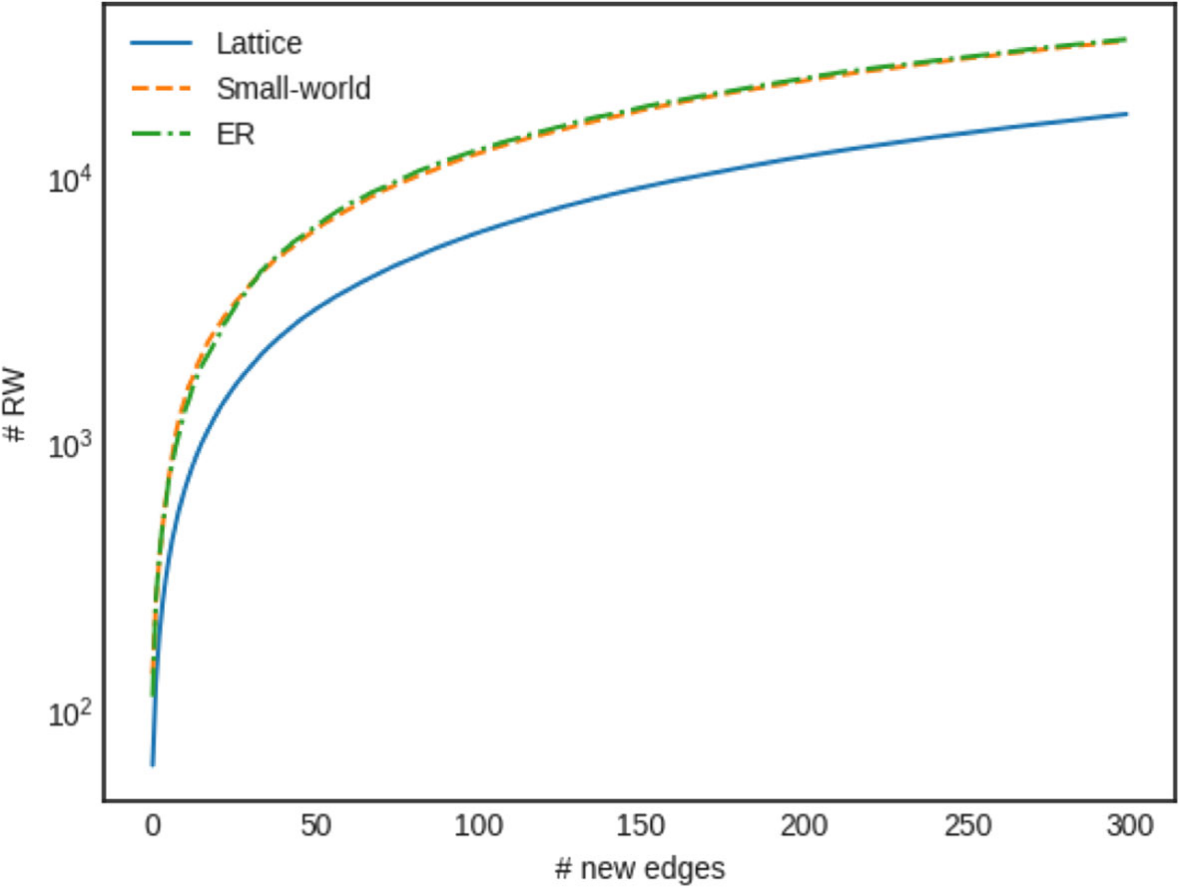
Effect of network topology (the axis of *#RW* affected is in logarithmic scale). As more new edges are added, more random walks are affected. The effect is more stressed in the case of the *Small-world* and *Erdos-Reyni* networks, than the *Lattice* network

We evaluate the effect of randomly adding a number of new edges in networks of different topologies, but same size. For each case, we report the number of the random walks that need to be updated. Figure 5 shows the results, where it becomes clear that as more new edges are added, more random walks are affected. The effect is more stressed in the case of the *Small-world* and *Erdos-Reyni* networks. This is to be expected, since these networks are known to have small diameter, therefore every node is easily accessible from any other node. As a result, every node has a high chance to appear in any random walk. In contrast, *Lattices* are known to have larger diameter, therefore only a small number of nodes (out of all nodes in the network) can be accessible by any random walk. As a result, nodes are more equally distributed in all random walks.

### Q2 effect of arriving edge importance

By answering **Q1**, it becomes evident that even a single new edge can have a dramatic effect in the number of random walks that need to be updated. Eventually, the number of random walks affected, will have an effect to the time performance of updating these random walks in our framework. In this set of experiments we perform a systematic analysis of the effect of the importance of an arriving edge to the time required for the update to occur. Importance of an incoming edge $e_{{ij}}^{t+1} = (n_{i}, n_j)$ at time *t*+1 in a network can be defined in different ways. Here, we define three metrics of edge importance, based on properties of the **endpoints***n*_*i*_,*n*_*j*_ of the arriving edge:

- *Sum of frequencies of edge endpoints inRW*_*t*_.
- *Sum of the node degrees of edge endpoints inG*_*t*_.
- *Sum of the node-betweenness of edge endpoints inG*_*t*_.

**Fig. 6:**
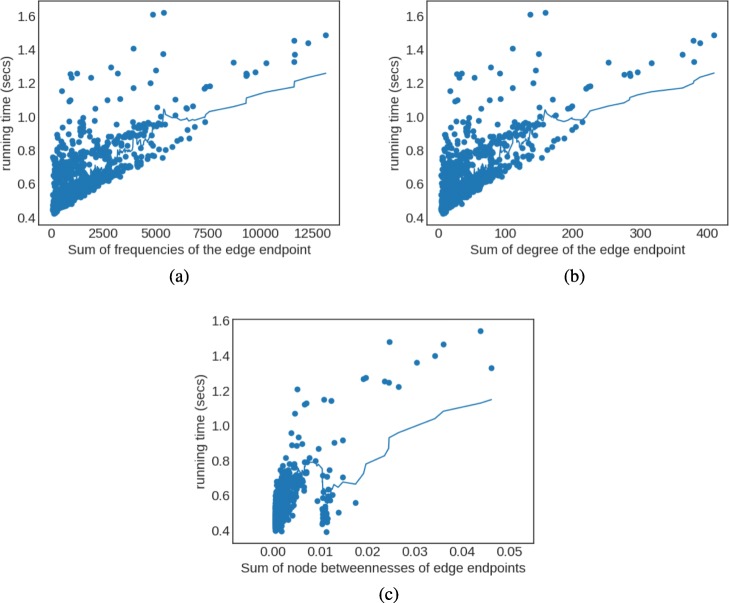
Dependency of EVONRL running time on importance of added edge as described by various metrics on PPI Network. **a** frequency of the new edge endpoints, **b** node degree of the new edge endpoints, and **c** node betweenness of the new edge endpoints

Results of the different experiments are presented in Fig. 6. The first observation is that important incoming edges are more expensive to update, sometimes up to three or four times (1.6sec vs 0.4sec). This is expected, as more random walks need to be updated. However, the majority of the edges are of least importance (lower left dense areas in Fig. 6a, b, and c), so fast updates are more common. Finally, the behavior of sum of node frequencies (Fig. 6a) and sum of node degrees (Fig. 6b) of the edge endpoints are correlated. This is because the node degree is known to be directly related to the number of random walks that traverse it. On the other hand, node-betweenness demonstrates more unstable behavior since it is mostly related to shortest paths and not just paths (which are related to random walks).

### Q3 accuracy performance of EVONRL

In this set of experiments we evaluate the accuracy performance of EVONRL and show that it is very **accurate**. At this point, it is important to note that evidence of our EVONRL performing well is provided by demonstrating it obtains **similar representations** to the ground truth provided by running StaticNRL on different instances of the evolving network. This is because the objective of our method is to resemble as much as possible what the actual changes in the original network are by incrementally maintaining a set of random walks and monitoring the changes. In practice, we aim to show that our proposed algorithm is able to update random walks in reserve such that they are always representing unbiased random walks that could have been obtained by running StaticNRL on the updated network. In these experiments, we show the representation learned by EvoNRL and the ground truth provided by the StaticNRL are similar to each other by using *a representational similarity* metric.

#### Similarity of two representations

Our goal here is to compare the representations learned by the neural network and show that EvoNRL results in a similar representations to ground truth provided by StaticNRL methods. Comparing representations in neural networks is difficult as the representations vary even across the neural networks trained on the same input data with the same task (Raghu et al. 2017). In this paper, representations are weights of the representation learned by either our EvoNRL method or the StaticNRL method, and they represent the representation learned by a skip-gram neural network. In order to determine the correspondence between these representations, we use the recent similarity measures of neural networks studied in (Morcos et al. 2018) and (Kornblith et al. 2019). Dynamics of neural networks call for a similarity metric that is *invariant to orthogonal transformation* and *invariant to isotropic scaling*. Assuming two representations $X \in \mathbb {R}^{n \times d}$ and $Y \in \mathbb {R}^{n \times d}$, we are concerned about a scalar similarity index *s*(*X*,*Y*) which can be used to compare the two neural network representations. There are many methods for comparing two finite set of vectors and measure the similarity between them. The simplest approach is to employ a dot-product based similarity. By summing the square dot-product of each corresponding pair of vectors in *X* and *Y*, we can have a similarity index between matrices *X* and *Y*. This approach is not practical as representations of the neural networks can be described on two different basis and result in a misleadingly similarity index. Therefore invariance to linear transforms is crucial in neural network representational similarity metrics. Recently, Canonical Correlation Analysis (CCA) (Hotelling 1992) is used as a tool to compare representations across networks. Canonical Correlation Analysis has been widely used to evaluate the similarity between computing models and brain activity. CCA can find similarity between representations where they are superficially dissimilar. Its invariance to linear transforms makes CCA a useful tool to quantify the similarity of EvoNRL and StaticNRL representations (Morcos et al. 2018).

**Canonical correlation analysis (CCA)**: Canonical Correlation Analysis (Hotelling 1992) is a statistical technique to measure the linear relationship between two multidimensional set of vectors. Ordinary Correlation analysis is highly dependent on the basis which the vectors are described on. The important property of CCA is that it is invariant to affine transformations of the variables which makes it a proper tool to measure representation similarity by. If we have two sets of matrices $X \in \mathbb {R}^{n \times d}$ and $Y \in \mathbb {R}^{n \times d}$, Canonical Correlation Analysis will find two bases, one for *X* and one for *Y* such that after their projections into these bases, their correlation will be maximized. for 1≤*i*≤*d*, the *i*^*th*^, canonical correlation coefficient is given by:

8 $$ \begin{aligned} \rho_{i} = \max_{w^{i}_{X}, w^{i}_{Y}} corr\left(Xw^{i}_{X}, Yw^{i}_{Y}\right)\\ {subject to} \; \forall_{j < i} \: Xw^{i}_{X} \bot Xw^{j}_{X}\\ \forall_{j < i} \: Yw^{i}_{Y} \bot Yw^{j}_{Y} \end{aligned}  $$

where the vectors $w^{i}_{X} \in \mathbb {R}^{d}$ and $w^{i}_{Y} \in \mathbb {R}^{d}$ transform the original matrices into canonical variables $Xw^{i}_{X}$ and $Yw^{i}_{Y}$.

9 $$ R^{2}_{{CCA}} = \frac{\Sigma_{i=1}^{d} \rho_{i}^{2}}{d}  $$

The mean squared CCA correlation (J. Ramsay et al. 1984), $R^{2}_{{CCA}}$ reports the sum of the squared canonical correlations. This sum is a metric that shows the similarity of the two multidimensional sets of vector.

**Experimental scenario**: In these experiments, the original network is the initial network at the beginning. We simulate random walks on this network and learn its representation. After that, we sequentially make changes (add edges, remove edges, add nodes and remove nodes) to the initial network and keep the random walks updated using EvoNRL. In certain points (for example after every 1000 edge addition in the PPI network), we learn the network representation in two ways. One is by simulating new random walks on the updated network (original network with new edges/nodes or missing edges/nodes) and second is learning the representation using EvoNRL. Now we have two representations of the same network and the goal is to compare them to see how similar EvoNRL is to StaticNRL. Note that StaticNRL simulates walks on the updated networks while EvoNRL has been updating the original random walk set. Representations obtained by StaticNRL are results of simulating random walks on the network. Because of the randomness involved in the process, it is typical that two differnet StaticNRL representations of the same network are not identical. We can measure, the similarity of the different representations using CCA. In our evaluation, we aim to demonstrate that EvoNRL is as similar to StaticNRL and that this similarity is comparable to the similarity obtained by applying StaticNRL multiple times on the same network. At any stage of the change (edge addition, edge deletion, node addition, node deletion) in the network, EvoNRL is updating the random walk set in a way that it is representing the network. First, we run StaticNRL multiple times (x5) on a network. Each StaticNRL is simulating a random walk set on the evolving network at certain times. Representations are two finite sets of vectors in *d*-dimensional space and compare how similar these two sets are.

**Fig. 7:**
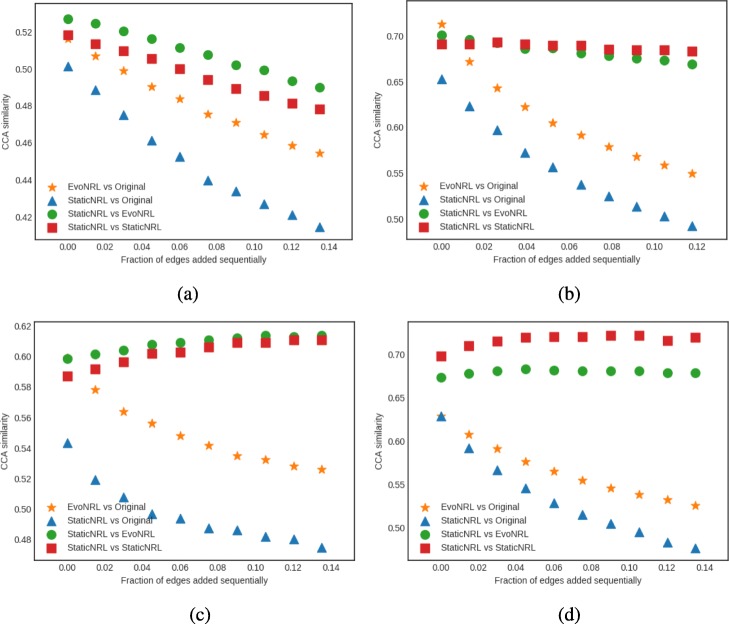
Accuracy performance of EVONRL — adding edges. **a** BlogCatalog, **b** PPI, **c** Facebook, **d** Cit-HepTh

*Adding edges*: Given a network *G*=(*V*,*E*), we can add a new edge by randomly picking two nodes in the network that are not currently connected and connect them. Adding new edges to the network should have an effect on the network embedding. By adding edges, as the network diverges from its original state, the embedding will diverge from the original network as well. Figure 7 shows the accuracy results of EvoNRL. We observe that the CCA similarity index of EVONRL follows the same trend as the StaticNRL in all the networks: BlogCatalog (Fig. 7a) and the PPI (Fig. 7b), Facebook (Fig. 7c) and Cit-HepTh (Fig. 7d) networks. The similarity of the two methods remains consistent as more edges are added (up to 12% of the number of edges in the original PPI; up to 14% of the number of edges in the original BlogCatalog, Facebook and Cit-HepTh). In Fig. 7, there are two sorts of comparison. First, The similarity of EvoNRL and the Original Network (The network before changes occur to it) is measured. The decreasing trend in orange stars in Fig. 7 shows that the EvoNRL is updating the set of random walks and the representations of the updated networks are diverging from the representation of the original network. On the other hand, we see that EvoNRL is more correlated to the original set of the random walk (orange stars), compared to StaticNRL (Blue Triangles). Blue Triangles are the average of canonical correlation of the original network with 4 different runs of StaticNRL. It shows that the representation of the evolving network is diverging from the original network. So far we have showed that EvoNRL is consistently updating the original set of random walks and makes difference in the network’s representation. The question is are these updates accurate? To answer this question we add edges step by step to the original network. Using EvoNRL we keep updating a set of random walk and get the representation of the network in a certain points. On the other hand, we run StaticNRL on the updated network at the same certain points. Because of the randomness of the random walks we repeat StaticNRL 4 times. We compare the StaticNRL representations obtained from the same network with each other to have a baseline of the similarity metric. The red squares showing as ’StaticNRL vs StaticNRL’ in Fig. 7 are showing the average similarity of representations of StaticNRL compared to each other 2 by 2. Our goal is to show, EvoNRL keeps updating the random walk set in an accurate way and the representation obtained by EvoNRL is as accurate as StaticNRL. To show this, we measure the canonical correlation of EvoNRL representation and the StaticNRl. We observe that (green circles) EvoNRL representations is very similar to the StaticNRL representations and can be an instance on StaticNRL.

**Fig. 8:**
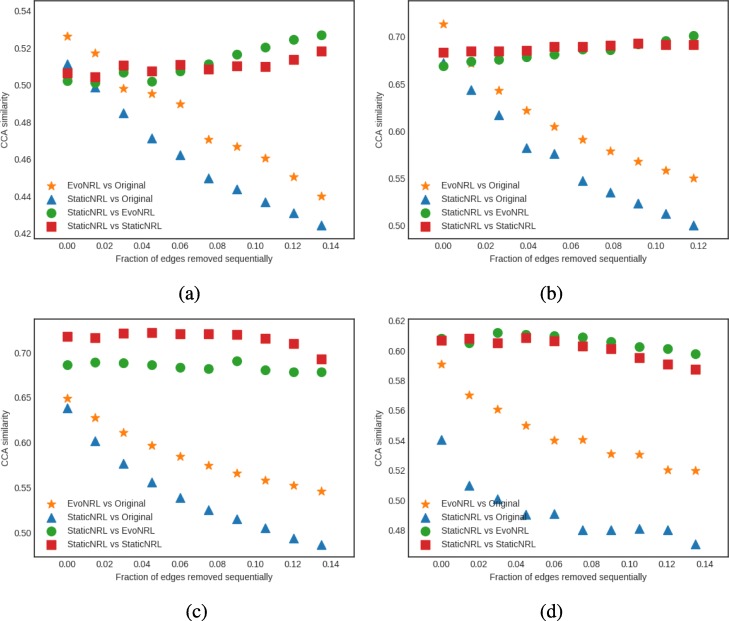
Accuracy performance of EVONRL — removing edges. **a** BlogCatalog, **b** PPI, **c** Cit-HepTh, **d** Facebook

*Removing edges*: Given a network *G*=(*V*,*E*), we can remove an edge by randomly choosing an existing edge *e*∈*E* and remove it from the network. Removing existent edges should have an effect in the network embedding. Figure 8 show the accuracy results of edge deletion. Similar to edge addition, We observe that the CCA similarity of EVONRL follows the same trend as the StaticNRL in all the networks: BlogCatalog (Fig. 8a) and the PPI (Fig. 8b), Facebook (Fig. 8c) and Cit-HepTh (Fig. 8d) networks.

**Fig. 9:**
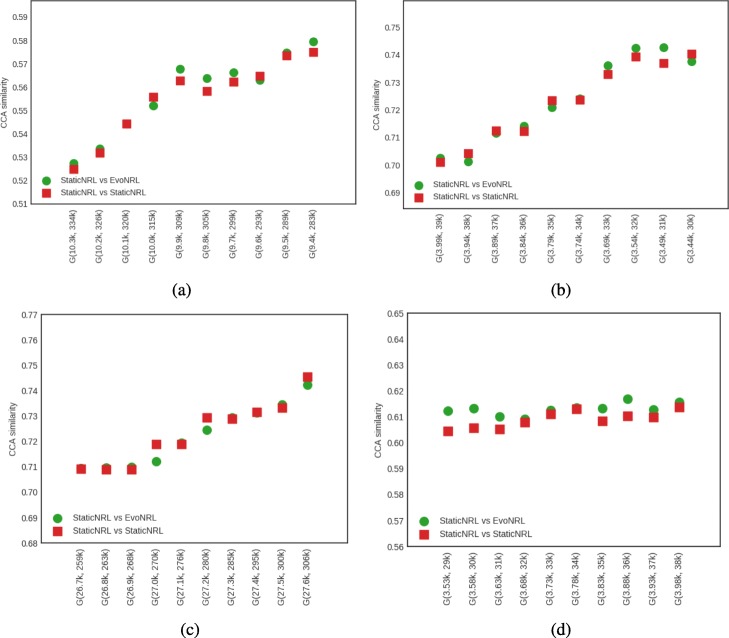
Accuracy performance of EVONRL — adding nodes. **a** BlogCatalog, **b** PPI, **c** Cit-HepTh, **d** Facebook

*Adding nodes*: As we described in “Evolving network representation learning” section node addition can be treated as a special case of edge addition. This is because whenever a node is added in a network, a number of edges attached to that node need to be added as well. To emulate this process, given a network *G*=(*V*,*E*), first we create a network *G*^′^=(*V*^′^,*E*^′^), where *V*^′^⊆*V*,*E*^′^⊆*E* as follows. We uniformly at random sample nodes *V*^′^⊆*V* from *G* and then remove these nodes and all their attached edges *E*^′^⊆*E* from *G*, forming *G*^′^. Following that process, we obtain a new network for BlogCatalog with *V*^′^=8312 and a new network for PPI with *V*^′^=3390 nodes, respectively. Then, we start adding the nodes *v*∈*V*^′′^=*V*∖*V*^′^ that have been removed from *G*, one by one. Whenever, a node *v*∈*V*^′′^ is added to *G*^′^, any edge between *v* and nodes existing in the current state of network *G*^′^ are added as well. Adding nodes to the network should have an effect in the network embedding. Figure 9 shows the accuracy results of node addition. CCA compares two sets of vectors with the same cardinality. Because the number of the nodes and therefore the number of the vectors in the representation are variant, we can not compare the updated representations with the original network. In these experiments we show that EvoNRL and StaticNRL on the same network are very similar to each other and EvoNRL is an accurate instance of StaticNRL.

**Fig. 10:**
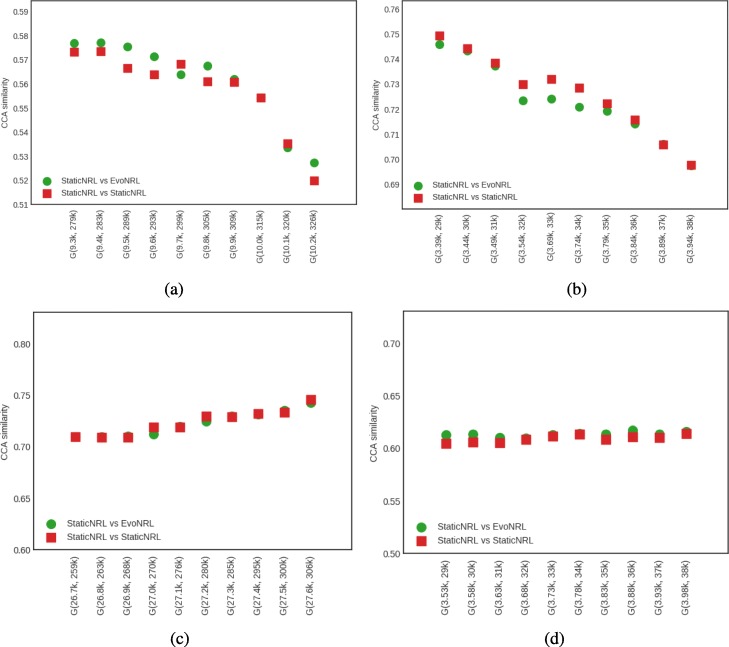
Accuracy performance of EVONRL — removing nodes. **a** BlogCatalog, **b** PPI, **c** Cit-HepTh, **d** Facebook

*Removing nodes*: As we described in “Evolving network representation learning” section node deletion can be treated as a special case of edge deletion. Given a network *G*=(*V*,*E*), we start removing nodes *v*∈*V* from the network, one by one. When a node is removed all the edges connecting this node to the network are removed as well. The process of removing nodes will result in a new network *G*^′^(*V*^′^,*E*^′^), where *V*^′^⊆*V* and *E*^′^⊆*E*. Removing existing nodes from the networ effect in the network embedding. Figure 10 shows the accuracy result of node deletion. In the evolving network, nodes are removed from the network sequentially and EvoNRL always maintains a valid set of random walks. we show that the representations obtained from these random walks are similar to StaticNRL representations. Same as node addition, because the number of the nodes are changing, we can not compare the representations with the original network’s representation. The experiments above provides strong evidence that our random walk updates are correct and can incrementally maintain a set of random walks that is their corresponding representations are similar to that of obtained by StaticNRL.

### Q4 classification performance of EVONRL

In this set of experiments we evaluate the accuracy performance of EVONRL and show that it is very **accurate**. At this point, it is important to note that evidence of our EVONRL performing well is provided by demonstrating it has **similar accuracy** to StaticNRL, for the various aspects of the evaluation (and **not** by demonstrating loss/gains in accuracy). This is because the objective of our method is to resemble as much as possible what the actual changes in the original network are by incrementally maintaining a set of random walks and monitoring the changes. In practice, we aim to show that our proposed algorithm is able to update random walks in reserve such that they are always representing unbiased random walks that could have been obtained by running StaticNRL on the updated network.

**Experimental scenario**: To evaluate our random walk update algorithm, we resort to accuracy experiments performed on a downstream data mining task: *multi-label classification*. The network topology of many real-world networks can change over time due to either adding/removing edges or adding/removing nodes in the network. In our experimental scenario, given a network we simulate and monitor network topology changes. Then, we run StaticNRL multiple times, one time after each network change and learn multiple network representations over time. The same process is followed for EVONRL but this time we only need to update the random walks *RW*_*t*_ at each time *t* and use these for learning multiple network representations over time. In multi-label classification each node has one or more labels from a finite set of labels. In our experiments, we see 50% of nodes and their labels in the training phase and the goal is to predict labels of the rest of the nodes. We use node vector representations as input to a one-vs-rest logistic regression classifier with L2 regularization. Finally, we report the *Macro*−*F*_1_ accuracy of the multi-label classification of StaticNRL and EVONRL as a function of the fraction of the network changes. For StaticNRL, since it is sensitive to the fresh set of random walks obtained every time, we run multiple times (10x) and report the averages. We experiment with the *BlogCatalog* and *PPI* networks. In the following paragraphs we present and discuss the results for each of the interesting cases (adding/removing edges, adding/removing nodes).

**Fig. 11:**
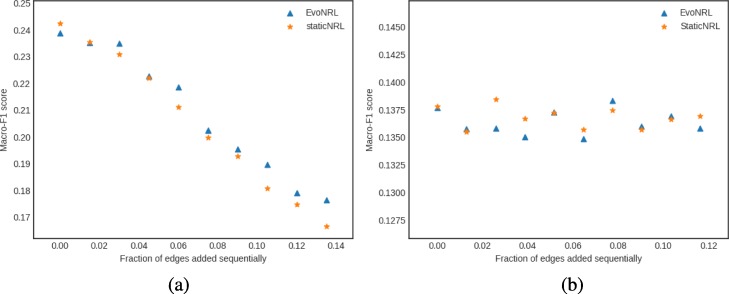
Accuracy performance of EVONRL — adding new edges. **a** BlogCatalog, **b** PPI

*Adding edges*: Given a network *G*=(*V*,*E*), we can add a new edge by randomly picking two nodes in the network that are not currently connected and connect them. Adding new edges to the network should have an effect on the network embedding and thus in the overall accuracy of the classification results. Figure 7 shows the results. We observe that the Macro-F_1_ accuracy of EVONRL follows the same trend as the one of StaticNRL in both the BlogCatalog (Fig. 11a) and the PPI (Fig. 11b) networks. The accuracy of the two methods remains consistent as more edges are added (up to 12% of the number of edges in the original PPI; up to 14% of the number of edges in the original BlogCatalog). This provides strong evidence that our random walk updates are correct and can incrementally maintain a set of random walks that is similar to that obtained by StaticNRL when applied in an updated network.

**Fig. 12:**
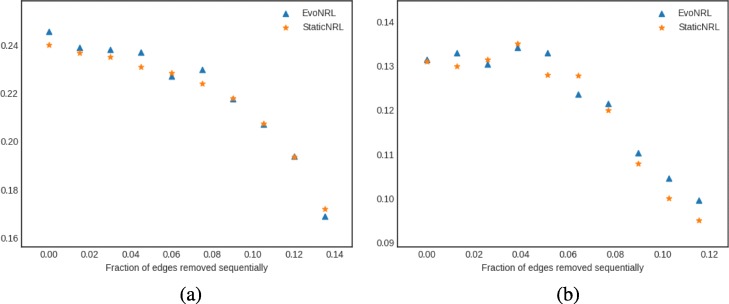
Accuracy performance of EVONRL — removing edges. **a** BlogCatalog, **b** PPI

*Removing edges*: Given a network *G*=(*V*,*E*), we can remove an edge by randomly choosing an existing edge *e*∈*E* and remove it from the network. Removing existent edges should have an effect in the network embedding and thus in the overall accuracy of the classification results. We evaluate the random walk update algorithm for the case of edge deletion in a way similar to that of adding edges. The only difference is that every time an edge is deleted at *t* we update random walks to obtain *RW*_*t*_. Then, the updated *RW*_*t*_ can be used for obtaining a network representation. Same setting is used in multi-label classification. Figure 12 shows the results. Again we observe that the Macro-F_1_ accuracy of EVONRL follows the same trend as the one of StaticNRL in both the BlogCatalog (Fig. 12a) and the PPI (Fig. 12b) networks.

**Fig. 13:**
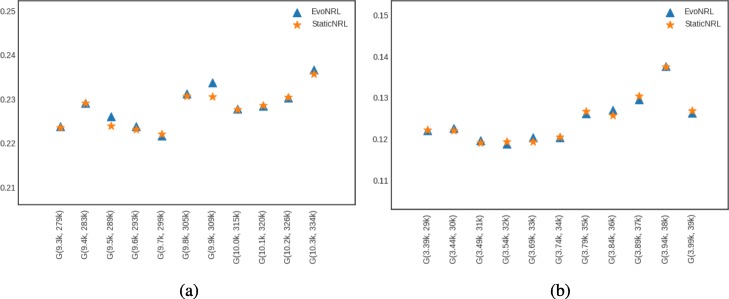
Accuracy performance of EVONRL — adding new nodes. **a** BlogCatalog, **b** PPI

*Adding nodes*: As we described in “Evolving network representation learning” section node addition can be treated as a special case of edge addition. This is because whenever a node is added in a network, a number of edges attached to that node need to be added as well. To emulate this process, given a network *G*=(*V*,*E*), first we create a network *G*^′^=(*V*^′^,*E*^′^), where *V*^′^⊆*V*,*E*^′^⊆*E* as follows. We uniformly at random sample nodes *V*^′^⊆*V* from *G* and then remove these nodes and all their attached edges *E*^′^⊆*E* from *G*, forming *G*^′^. Following that process, we obtain a new network for BlogCatalog with *V*^′^=8312 and a new network for PPI with *V*^′^=3390 nodes, respectively. Then, we start adding the nodes *v*∈*V*^′′^=*V*∖*V*^′^ that have been removed from *G*, one by one. Whenever, a node *v*∈*V*^′′^ is added to *G*^′^, any edge between *v* and nodes existing in the current state of network *G*^′^ are added as well. Adding nodes to the network should have an effect in the network embedding and thus in the overall accuracy of the classification results. We evaluate the random walk update algorithm for the case of node addition in a way similar to that of adding edges. The only difference is that every time a node is added at *t* we update random walks to obtain *RW*_*t*_, by adding a number of edges. Then, the updated *RW*_*t*_ can be used for obtaining a network representation. Figure 13 shows the results. Again we observe that the Macro-F_1_ accuracy of EVONRL follows the same trend as the one of StaticNRL in both the BlogCatalog (Fig. 13a) and the PPI (Fig. 13b) networks.

**Fig. 14:**
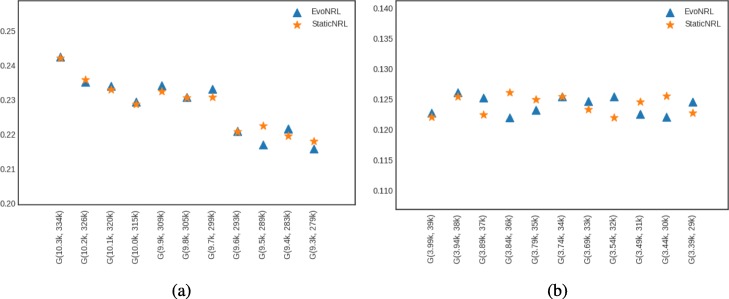
Accuracy performance of EVONRL — removing new nodes. **a** BlogCatalog, **b** PPI

*Removing nodes*: As we described in “Evolving network representation learning” section node deletion can be treated as a special case of edge deletion. Given a network *G*=(*V*,*E*), we start removing nodes *v*∈*V* from the network, one by one. When a node is removed all the edges connecting this node to the network are removed as well. The process of removing nodes will result in a new network *G*^′^(*V*^′^,*E*^′^), where *V*^′^⊆*V* and *E*^′^⊆*E*. Removing existing nodes from the network should have an effect in the network embedding and thus in the overall accuracy of the classification results. We evaluate the random walk update algorithm for the case of node deletion in a way similar to that of deleting edges. The only difference is that every time a node is deleted at *t* we update random walks to obtain *RW*_*t*_, by removing a number of edges. Then, the updated *RW*_*t*_ can be used for obtaining a network representation. Figure 14 shows the results. Again we observe that the Macro-F_1_ accuracy of EVONRL follows the same trend as the one of StaticNRL in both the BlogCatalog (Fig. 14a) and the PPI (Fig. 14b) networks.

**Fig. 15:**
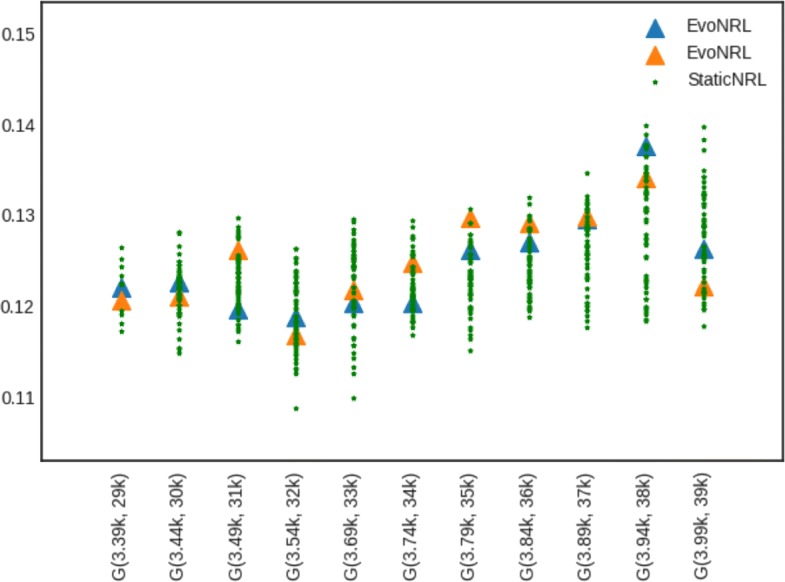
Accuracy values obtained by running StaticNRL multiple times on the same network. The values are significantly fluctuating due to sensitivity to the set of random walks obtained. Similarly, EVONRL is sensitive to the initial set of random walks obtained. Two instances of EVONRL are shown, each of which operates on a different initial set of random walks

**Discussion about accuracy value fluctuations**: While we have demonstrated that EVONRL is able to resemble the accuracy performance obtained by StaticNRL, one can observe that in some cases the accuracy values of the methods can substantially fluctuate. This behavior can be explained by the sensitivity of the StaticNRL methods to the set of random walks obtained from the network, as discussed in the motivating example of “Evaluation of the stability of StaticNRL methods” section. EVONRL would also inherit this problem, as it depends on an initially obtained set of random walks that is subsequently updated at every network topology change. To demonstrate this sensitivity effect, we run control experiments on the PPI network for the case of adding new nodes in the network *G*, similar to the experiment in Fig. 13b. However, this time, instead of reporting the average over a number of runs for the StaticNRL method, we report all its instances (ref(Fig. 15)). In particular, as we add more nodes (the number of nodes increases from 3390 to 3990) a new network is obtained. We report the accuracy values obtained by running StaticNRL multiple times (40x) on the same network. We also depict the values of two different runs for EVONRL. Each run obtains an initial set of random walks that is incrementally updated in subsequent network topology changes. It becomes evident that the StaticNRL values can significantly fluctuate due to the sensitivity to the set of random walks obtained. It is important to note that EVONRL manages to fall within the range of these fluctuations.

### Q5 time performance of EVONRL

**Fig. 16:**
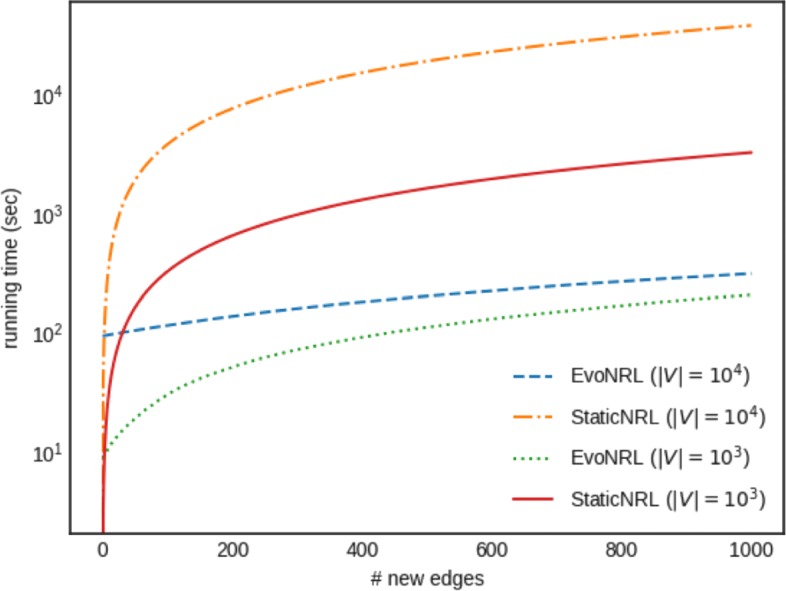
EVONRL scalability (running time axis is in logarithmic scale). StaticNRL scales linearly to the number of new edges added in the network, since it has to run again and again for every new edge. At the same time, EVONRL is able to accommodate the changes more than 100 times faster than StaticNRL. This behavior is even more stressed in the larger network (where the number of nodes is larger)

In this set of experiments we evaluate the time performance of our method and show that EVONRL is very **fast**. We run experiments on two *Small-world* networks (Watts-Strogatz (*p*=0.5)), with two different number of nodes (|*V*|=1000 and |*V*|=10000). We evaluate EVONRL against a standard StaticNRL method from the literature (Grover and Leskovec 2016). Both algorithms start with the same set of random walks *RW*. As new edges are arriving, StaticNRL needs to learn a new network representation by resimulating a new set of walks every time. On the other hand, EVONRL has the overhead of first indexing the set of initial random walks *RW*. Then, for every new edge that is arriving it just needs to perform the necessary updates as described earlier. Figure 16 shows the results. It can be seen that the performance of StaticNRL is linear to the number of new edges, since it has to run again and again for every new edge. At the same time, EVONRL is able to accommodate the changes more than 100 times faster than StaticNRL. This behavior is even more stressed in the larger network (where the number of nodes is larger). By increasing the number of nodes, running StaticNRL becomes significantly slower, because by design it needs to simulate larger amount of random walks. On the other hand, EVONRL has a larger initialization overhead, but after that it can easily accommodate new edges. This is because every update is only related to the number of random walks affected and not the size of the network. This is an important observation, as it means that the benefit of EVONRL will be more stressed in larger networks.

### Q6 decision-making performance of EVONRL

**Fig. 17:**
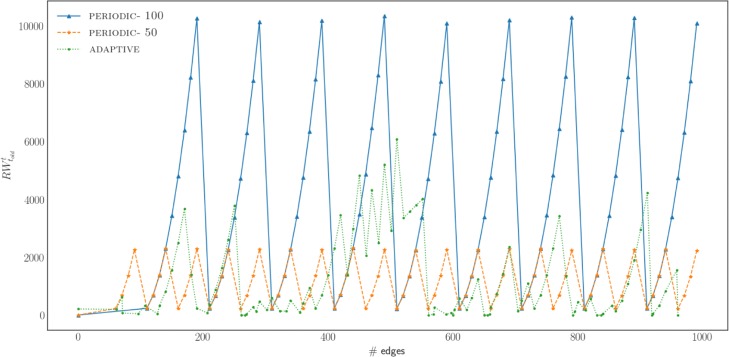
Comparative analysis of different strategies for determining when to obtain a network representation. The PERIODIC methods will obtain a new representation every 50 or 100 time steps (i.e., network changes). Our proposed method, ADAPTIVE, is combining a *peak detection* method and a *cumulative changes* cut-off method to determine the time to obtain a new network representation. As a result it is able to make more informed decisions and perform better. This is depicted by smaller (on average) changes of the $RW_{t_{old}}^{t}$, which implies that a more accurate network representation is available for down-stream network mining tasks

In this experiment, we compare the two different strategies for deciding when to obtain a network representation, PERIODIC and ADAPTIVE. The experiment is performed using the *BlogCatalog* network and the changes in the network are related to edge addition. For presentation purposes, we limit the experiment to 1000 edges. The evaluation of this experiment is based on the number of random walk changes $RW^{t}_{t_{{old}}}$ between a random walk set obtained at time *t* (one edge is added at each time) and a previously obtained network representation as defined by each strategy. Results are shown in Fig. 17. The PERIODIC strategy represents a “blind” strategy where new embeddings are obtained periodically (every 50 times steps or every 100 time steps). On the other hand, the ADAPTIVE method is able to make informed decisions as it monitors the importance of every edge added in the network. The ADAPTIVE method is basing its decisions on the a peak detection method (*τ*=3.5) and a method that monitors cumulative effects due to a number of changes (*cutoff*=4000). As a result, ADAPTIVE is able to perform much better, as depicted by many very low values in the $RW^{t}_{t_{{old}}}$.

## Extensions and variants

While our algorithms have been described and evaluated on a single machine, they have been designed with scalability in mind. Recall that our indexing and searching of random walks is supported by Elasticsearch^5^, which itself is based on Apache Lucene^6^. Elasticsearch is a distributed index and search engine that can naturally scale to very large number of documents (i.e., a very large number of random walks in our setting). There are a couple of basic concepts that make a distributed index and search engine scalable enough to be suitable for the needs of our problem:

- *Index sharding*: One of the great features of a distributed index is that it’s designed from the ground up to be *horizontally scalable*, meaning that more nodes can be added to the cluster to match the capacity required by the problem. It achieves horizontal scalability by *sharding* its index and assigning each shard to a node in the cluster. This allows each node to have to deal with only part of the full random walk index. Furthermore, it also has the concept of *replicas* (copies of shards) to allow fault tolerance and redundancy, as well as an increased throughput.
- *Distributed search*: Searching a distributed index is done in two phases:
  - *Querying*: Each query *q* is sent to all shards of the distributed index and each shard returns a list of the matching random walks. Then, the lists are merged, sorted and returned along with the *random walk ids*.
  - *Fetching*: Each random walk is fetched by the shard that owns it using the *random walk id* information. Random walks that lie in different shards can be processed in parallel by the method requesting them.

Therefore, while our algorithms are demonstrated in smaller networks for clarity of coverage and better representation of the algorithmic comparison, in practice they can be easily and naturally expanded to very large graphs. Extensions of the algorithms to a distributed environment are out of the scope of this work.

## Related work

Our work is mostly related to research in the area of *static network representations learning* and *dynamic network representation learning*. It is also related to research in *random walks*.

**Static network representations learning**: Starting with Deepwalk (Perozzi et al. 2014), these methods use finite length random walks as their sampling strategy and inspired by word2vec (Mikolov et al. 2013b) use skip-gram model to maximize likelihood of observing a node’s neighborhood given its low dimensional vector. This neighborhood is based on random walks. LINE (Tang et al. 2015) proposes a breadth-first sampling strategy which captures first-order proximity of nodes. In (Grover and Leskovec 2016), authors presented *node*2*vec* that combines LINE and Deepwalk as it provides a flexible control of random walk sampling strategy. HARP (Chen et al. 2017) extends random walks by performing them in a repeated hierarchical manner. Also there have been further extensions to the random walk embeddings by generalizing either the embeddings or random walks (Chamberlain et al. 2017*;*Perozzi et al. 2016). Role2Vec (Ahmed et al. 2018) maps nodes to their type-functions and generalizes other random walk based embeddings. Our work is focusing on how many of the above methods introduced for static networks (the ones that use random walks) can be extended to the case of evolving networks.

**Dynamic network representation learning**: Existing work on embedding dynamic networks often apply static embedding to each snapshot of the network and then rotationally align the static embedding across each time-stamp (Hamilton et al. 2016). Graph factorization approaches attempted to learn the embedding of dynamic graphs by explicitly smoothing over consecutive snapshots (Ahmed et al. 2013). DANE (Li et al. 2017) is a dynamic attributed network representation framework which first proposes an offline embedding method, then updates the embedding results based on the changes in the attributed evolving network. Know-Evolve (Trivedi et al. 2017) proposes an evolving network embedding method in a knowledge-graph for entity embeddings based on multivariate event detection. EvoNRL is a more general method which extracts the network representation without using node features or explicit use of events. CTDN (Nguyen et al. 2018) is a random walk-based continuous-time dynamic network embedding. Our work is different from this paper in two aspects. First the random walk in CTDN is a temporal random walk and second CTDN is not an online framework and you need to have all the snapshots of the network before embedding it. HTNE (Zuo et al. 2018) tries to model the temporal network as a self-excited system and using Hawkes process model neighbourhood formation in the network and optimize the embedding based on point-time process. HTNE is an online dynamic network embedding framework which is different from EvoNRL as it uses history in its optimization and it needs to be tuned for history in each step. NetWalk (Yu et al. 2018) is a random walk based clique embedding. The random walk update in that paper is different from EvoNRL. First in NetWalk, the reservoir is in memory which finds the next step based on the reservoir and it doesn’t benefit the sampling method used in EvoNRL which is based on node degrees. Also, EvoNRL leverages the speed of the inverted-indexing tools. In (Du et al. 2018), authors propose a dynamic skip-gram framework which is orthogonal to our work. Moreover, (Rudolph and Blei 2018) proposes a dynamic word embedding which uses Gaussian random walks to project the vector representations of words over time. The random walks in that work are based on vector representations and are defined over time-series, which is different to our approach.

**Random walks**: Our work is also related to general concept of random walks on networks (Lovász 1993) and its applications (Craswell and Szummer 2007;Page et al. 1999). READS (Jiang et al. 2017) is an indexing scheme for Simrank computation in dynamic graphs which keeps an online set of reverse-random walks and re-simulates the walks on all of the instances of the node queries. Our proposed method, keeps a set of finite-length random walks which is different from pagerank random walks and has a different sampling strategy and application compared to READS. Another aspects of random walk used in streaming data are continuous-time random walks. Continuous Time Random Walks (CTRW) (Kenkre et al. 1973) are widely studied in time-series analysis and has applications in Finance (Paul and Baschnagel 2010). CTRW is orthogonal to our work as we are not using time-variant random walks and our random walks do not jump over time.

## Conclusions

Our focus in this paper is on learning representations of evolving networks. To extend static random walk based network representation methods to evolving networks, we proposed a general framework for updating random walks as new edges and nodes are arriving in the network. The updated random walks leverage time and space efficiency of inverted indexing methods. By indexing an initial set of random walks in the network and efficiently updating it based on the occurring network topology changes, we manage to always keep a valid set of random walks with minimum possible divergence from the initial random walk set. Our proposed method, EVONRL, utilizes the always valid set of random walks to obtain new network representations that respect the changes that occurred in the network. We demonstrated that our proposed method, EVONRL is both accurate and fast. We also discussed the interesting trade-off between time performance and accuracy when obtaining subsequent network representations. Determining the right time for obtaining a network embedding is a challenging problem. We demonstrated that simple strategies for monitoring the changes that occur in the network can provide support in decision making. Overall, the methods presented are easy to understand and simple to implement. They can also be easily adopted in diverse domains and applications of graph/network mining.

**Reproducibility:** We make source code and data sets used in the experiments publicly available^7^ to encourage reproducibility of results.

## Abbreviations

CCA: Canonical correlation analysis
Cit-HepTh: High energy physics theory citation network
CTDN: Continious-time dynamic network embedding
CTRW: Continuous time random walks
DANE: Dynamic attributed network embedding
DBLP: Digital bibliography &library project
EvoNRL: Evolving network representation learning
HOPE: High-order proximity preserved embedding
HTNE: Hawkes process based temporal network embedding
NSERC: Natural sciences and engineering research council of Canada PPI: Protein-protein interaction
READS: Randomized efficient accurate dynamic SimRank computation
StaticNRL: Static network representation learning
TADW: Text-associated DeepWalk

## Footnotes

1 node2vec — code is available at https://github.com/aditya-grover/node2vec

2 NumPy — https://www.numpy.org/

3 https://github.com/RaRe-Technologies/gensim

4 code is available at https://github.com/farzana0/EvoNRL

5 Elasticsearch: https://www.elastic.co

6 Apache Lucene: http://lucene.apache.org/core/

7 https://github.com/farzana0/EvoNRL
